# Dephosphorylated parafibromin is a transcriptional coactivator of the Wnt/Hedgehog/Notch pathways

**DOI:** 10.1038/ncomms12887

**Published:** 2016-09-21

**Authors:** Ippei Kikuchi, Atsushi Takahashi-Kanemitsu, Natsuki Sakiyama, Chao Tang, Pei-Jung Tang, Saori Noda, Kazuki Nakao, Hidetoshi Kassai, Toshiro Sato, Atsu Aiba, Masanori Hatakeyama

**Affiliations:** 1 Division of Microbiology, Graduate School of Medicine, The University of Tokyo, Tokyo 113-0033, Japan; 2 Laboratory of Animal Resources, Center for Disease Biology and Integrative Medicine, Graduate School of Medicine, The University of Tokyo, Tokyo 113-0033, Japan; 3 Division of Gastroenterology, Keio University School of Medicine, Tokyo 108-8345, Japan; 4 Max Planck-The University of Tokyo Center for Integrative Inflammology, Tokyo 113-0033, Japan; 5 CREST, Japan Science and Technology Agency, Saitama 332-0012, Japan

## Abstract

Evolutionally conserved Wnt, Hedgehog (Hh) and Notch morphogen pathways play essential roles in the development, homeostasis and pathogenesis of multicellular organisms. Nevertheless, mechanisms that intracellularly coordinate these signal inputs remain poorly understood. Here we found that parafibromin, a component of the PAF complex, competitively interacts with β-catenin and Gli1, thereby potentiating transactivation of Wnt- and Hh-target genes in a mutually exclusive manner. Parafibromin also binds to the Notch intracellular domain (NICD), enabling concerted activation of Wnt- and Notch-target genes. The transcriptional platform function of parafibromin is potentiated by tyrosine dephosphorylation, mediated by SHP2 phosphatase, while it is attenuated by tyrosine phosphorylation, mediated by PTK6 kinase. Consequently, acute loss of parafibromin in mice disorganizes the normal epithelial architecture of the intestine, which requires coordinated activation/inactivation of Wnt, Hh and/or Notch signalling. Parafibromin integrates and converts signals conveyed by these morphogen pathways into appropriate transcriptional outputs in a tyrosine phosphorylation/dephosphorylation-regulated manner.

## Abstract (web summary)

Normal epithelial intestine organisation requires Wnt and Hedgehog signalling activity. Here, the authors show that parafibromin can activate both pathways in a mutually exclusive manner and is important for intestinal homeostasis.

## Graphical abstract

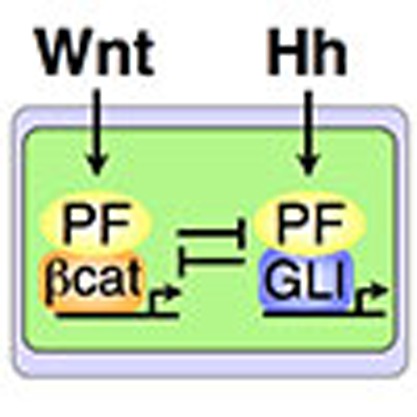

Morphogens act as positional information cues that determine cell fate specification in multicellular organisms^1^. Morphogens also act as mitogens to control cell proliferation and thereby regulate organ sizes^2^. Graded concentrations/availability of morphogens underlie differential gene expression, providing the mechanistic basis for cell fate-dependent growth and differentiation. Morphogens are secreted or cell surface protein ligands that bind to specific cell membrane receptors on target cells and initiate intracellular signal transduction cascades that culminate in post-translational regulation of the activities of transcriptional effectors that are specific to each signalling pathway^3^. The Wnt, Hedgehog (Hh) and Notch signalling pathways are among only a handful of evolutionally conserved morphogen pathways mediating embryonic development as well as homeostasis of adult tissues^4^,^5^,^6^. These morphogen signals play critical roles in the maintenance of cell stemness, commitment of cell differentiation, and expansion and differentiation of progenitor cells^4^,^5^,^6^. Although each morphogen pathways are independent linear conduits, they often cross-regulate at multiple steps during signal transmission to mutually influence signal magnitude, either positively or negatively, for regulation of the fate of individual cells.

Opposing roles of Wnt signalling and Hh signalling have been implicated in cell fate decision-making during embryogenesis and organogenesis. In the development and maintenance of the microarchitecture of intestinal epithelia, the Wnt signal is activated in the stem/progenitor cell compartment (crypt compartment), whereas Hh ligands, secreted from mature villus enterocytes, act on the sub-villus mesenchymal component^7^. Antagonistic roles of Wnt signalling and Hh signalling have also been reported in the dorso-ventral patterning of the developing central nervous system in vertebrates^8^. In neural tube development, Hh signalling acts as a determinant of ventral progenitor identity, whereas Wnt signalling functions as a determinant of dorsal progenitor identity. This ventralization activity of Hh signalling is antagonized by the Wnt pathway, at least in part through the induction of Gli3/GLI3, an inhibitor of the Hh pathway, by canonical Wnt signalling^9^. On the other hand, the Wnt signal acts together with the Notch signal in the crypt compartment of the intestine in determining cell fate, either intestinal stem cell or progenitor cell, most probably through the relative magnitude of activated Wnt signalling and Notch signalling^10^,^11^,^12^. Despite complicated and intertwined functional interplays among these morphogen pathways, however, very little is known about the mechanism through which a single cell perceives and integrates information made by two or more distinct morphogen gradients to generate appropriate transcriptional outputs.

Parafibromin was originally identified as a product of the *HRPT2* (*hyperparathyroidism-jaw tumour type 2*) gene, loss-of-function mutation of which is associated with hyperparathyroidism-jaw tumour syndrome^13^. Inactivating mutations in *HRPT2* have also been identified in sporadic parathyroid cancers^14^,^15^, indicating its tumour suppressive role in the parathyroid gland. Parafibromin is the human (mammalian) orthologue of budding yeast Cdc73, a component of the RNA Polymerase II (Pol II)-associated factor (PAF) complex^16^. The PAF complex, comprising Paf1, Ctr9, parafibromin/Cdc73, Rtf1 and Leo1, accompanies Pol II from the promoter to the mRNA termination site. In human cells, Ski8 is also a component of the PAF complex^17^. The PAF complex has been linked to transcription-related processes including communication with transcriptional activators, recruitment and activation of histone modification factors, facilitation of elongation on chromatin templates and recruitment of mRNA termination factors^18^. Absence of, or mutation in, the PAF components results in alterations in gene expression that can lead to deregulation of developmental programs and loss of control of cell growth and differentiation, thereby predisposing to various diseases including cancer^17^. Although human parafibromin and yeast Cdc73 share 32% amino-acid identity, parafibromin contains an N-terminal extension that is also present in Hyrax, the *Drosophila* orthologue of parafibromin, indicating that parafibromin/Hyrax has a metazoan-specific role via the N-terminal region^19^. Indeed, the N-terminal region of Hyrax was found to interact with β-catenin, the transcriptional effector of the canonical Wnt pathway, and to potentiate activation of Wnt-target genes^20^. Furthermore, the interaction between parafibromin and β-catenin is potentiated by tyrosine dephosphorylation of parafibromin on Tyr-290, Tyr-293 and Tyr-315 (Y290/293/315), which is mediated by the protein tyrosine phosphatase SHP2 in the nucleus^21^. Interestingly, translocalization of SHP2 from the cytoplasm to the nucleus is mediated by interaction with YAP or its homologue TAZ, target transcriptional coactivators of the tumour suppressive Hippo signalling pathway^22^. Since Hyrax is also involved in Hh signalling^23^, parafibromin/Hyrax may regulate multiple intracellular signalling pathways in metazoans.

In this study, we found that parafibromin acts as a nuclear platform/scaffold protein that intracellularly coordinates multiple morphogen signalling pathways. Parafibromin competitively interacted with the Wnt-signal effector β-catenin and the Hh-signal effector Gli1, thereby potentiating transactivation of Wnt-target genes and Hh-target genes in a mutually exclusive manner. Parafibromin also bound to the Notch-signal effector, Notch intracellular domain (NICD), and directed cooperative activation of Notch and Wnt signals. The platform function of parafibromin was strengthened by SHP2-mediated tyrosine dephosphorylation on Y290/293/315 but was attenuated by tyrosine phosphorylation on the same residues via protein tyrosine kinase 6 (PTK6; also known as breast tumour kinase (BRK)), which was induced in response to another morphogen, bone morphogenetic protein (BMP). This work unravelled a critical role of parafibromin in integrating multiple distinct morphogen signals and converting them to appropriate transcriptional outputs.

## Results

### Dephosphorylated parafibromin promotes the Hedgehog signal

**Figure 1:**
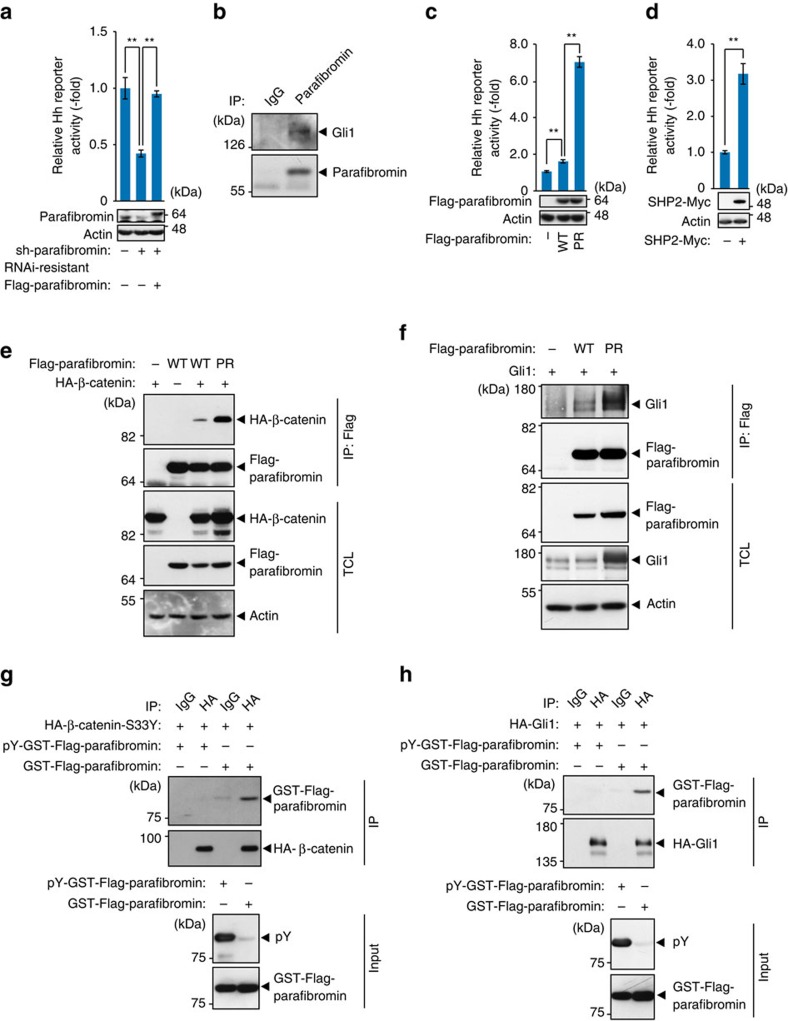
Parafibromin transactivates Hedgehog signalling in a tyrosine dephosphorylation-dependent manner. (**a**) Parafibromin knockdown results in reduced Hh signal activity. Hh-responsive luciferase reporter assays were performed in HEK293T cells. Total cell lysates (TCLs) were subjected to immunoblotting (*n*=3, mean±s.d. ^**^P<0.01; ANOVA with Bonferroni's *post hoc* test). (**b**) Parafibromin interacts with Gli1. TCLs prepared from SKES cells were sequentially immunoprecipitated (IP) with an anti-parafibromin antibody and immunoblotted. (**c**) Phospho-resistant (PR)-parafibromin shows increased ability to activate Hh signalling. Hh-responsive luciferase reporter assays were performed in HEK293T cells (*n*=3, mean±s.d. ^**^P<0.01; ANOVA with Bonferroni's *post hoc* test). (**d**) SHP2 expression promotes Hh signalling. Hh-responsive luciferase reporter assays were performed in A549 cells (*n*=3, mean±s.d. ^**^P<0.01; a two-tailed unpaired Student's *t*-test). (**e**,**f**) PR-parafibromin shows increased binding to β-catenin and Gli1. HEK293T cells were transfected with the indicated vectors. TCLs were sequentially immunoprecipitated (IP) and immunoblotted. (**g**,**h**) Non-phosphorylated parafibromin specifically interacts with β-catenin and Gli1. HEK293T cells were transfected with the indicated vectors. TCLs were immunoprecipitated with the indicated antibodies and immunoprecipitates (IP) were incubated with recombinant phosphorylated/non-phosphorylated parafibromin. The reaction mixtures were subjected to immunoblotting.

In a previous study*, Drosophila* parafibromin Hyrax has been reported to activate Hh signalling^23^. To confirm the role of parafibromin in the regulation of the mammalian Hh signal, we knocked down endogenous parafibromin by specific shRNA in HEK293T human embryonic kidney cells and performed a Hh-dependent luciferase reporter assay. Inhibition of parafibromin resulted in significant reduction of Hh-reporter activity, which was restored by ectopic expression of RNAi-resistant parafibromin (Fig. 1a), suggesting that parafibromin plays a positive role in the nuclear Hh signalling. We next performed a co-immunoprecipitation study using SKES human Ewing sarcoma cells, in which Hh signalling is constitutively activated^24^, and found that endogenous Gli1 was co-precipitated with endogenous parafibromin (Fig. 1b). These results indicated that parafibromin potentiated Hh signalling by interacting with the Hh effector Gli1, although the data did not discriminate whether the observed interaction is direct or whether it is indirect, being mediated by another cellular component.

Previously, we demonstrated that tyrosine dephosphorylation at Y290/293/315 of parafibromin potentiates parafibromin/β-catenin interaction and thereby enhances Wnt activation^21^. To determine whether the status of parafibromin tyrosine phosphorylation also influences the magnitude of Hh signal activation by parafibromin, we transiently expressed a phosphorylation-resistant (PR)-parafibromin mutant (parafibromin-Y290/293/315F), which mimics SHP2-dephosphorylated parafibromin^21^, and examined its role in Hh signal activation in HEK293T cells. As a result, PR-parafibromin activated the Hh reporter much more strongly than did wild-type parafibromin, either by single expression or co-expression with Gli1 (Fig. 1c and Supplementary Fig. 1a). Ectopically expressed SHP2 also enhanced Hh-reporter activation in A549 human lung carcinoma cells (Fig. 1d). These results collectively indicated that parafibromin potentiated Hh signalling in a tyrosine dephosphorylation-dependent manner as was the case of Wnt/β-catenin signalling. To gain an insight into the mechanism by which parafibromin strengthens the Hh signal, we investigated the effect of parafibromin tyrosine phosphorylation on Gli1 binding. In a co-precipitation experiment, PR-parafibromin exhibited a marked increase in the ability to form a complex with Gli1 compared with wild-type parafibromin as was the case of β-catenin (Fig. 1e,f), indicating that the interaction is strengthened upon tyrosine dephosphorylation of parafibromin. To determine if the observed Gli1 or β-catenin binding was strictly dependent on parafibromin dephosphorylation, we generated and purified recombinant parafibromin, either non-phosphorylated or tyrosine-phosphorylated by v-Src in *E. coli*, and performed an *in vitro* binding assay of parafibromin with Gli1 or β-catenin. As a result, non-phosphorylated parafibromin interacted with β-catenin or Gli1, whereas tyrosine-phosphorylated parafibromin did not (Fig. 1g,h). Thus, complex formation of parafibromin with β-catenin or Gli1 required parafibromin tyrosine dephosphorylation. From these observations, we concluded that parafibromin acts as a transcriptional coactivator of β-catenin and Gli1 in Wnt and Hh signalling, respectively, by forming a specific complex with the tyrosine-dephosphorylated form of parafibromin.

### Dephosphorylated parafibromin enhances Notch signalling

**Figure 2:**
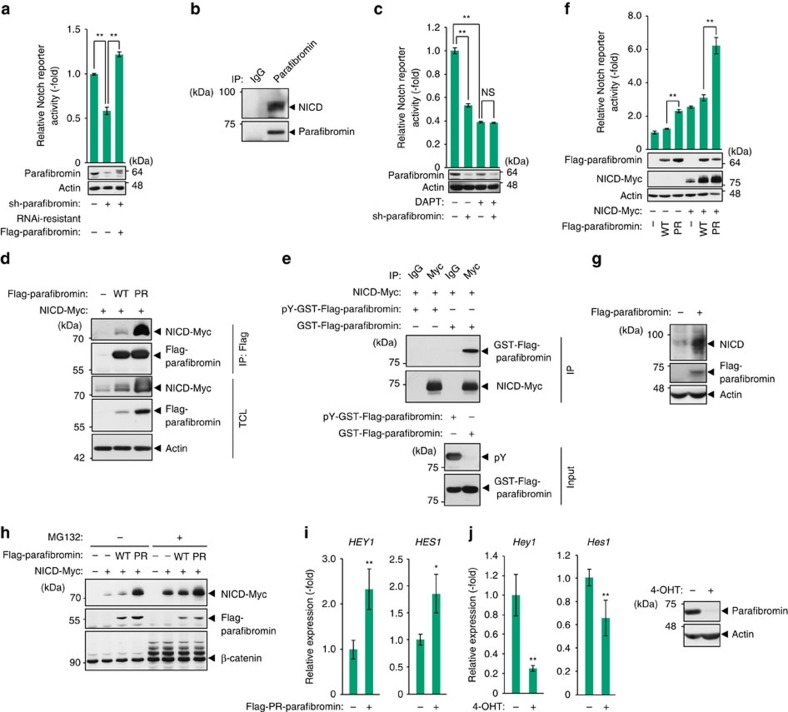
Parafibromin acts as a transcriptional coactivator in Notch signalling. (**a**) Parafibromin knockdown results in reduced Notch signal activity. Notch-responsive luciferase reporter assays were performed in HEK293T cells. Total cell lysates (TCLs) were subjected to immunoblotting (*n*=3, mean±s.d. ^**^P<0.01; ANOVA with Bonferroni's *post hoc* test). (**b**) Parafibromin forms a complex with NICD. TCLs prepared from MCF7 cells were sequentially immunoprecipitated (IP) with an anti-parafibromin antibody and immunoblotted. (**c**) A γ-secretase inhibitor (DAPT) treatment blocked the parafibromin-dependent Notch reporter activation. Notch-responsive luciferase reporter assays were performed in HEK293T cells. Cells were incubated with medium containing 100 μM DAPT for 48 h (*n*=3, mean±s.d. ^**^P<0.01; ANOVA with Bonferroni's *post hoc* test). (**d**) Phospho-resistant (PR)-parafibromin shows increased NICD binding. HEK293T cells were transfected with the indicated vectors. TCLs were sequentially immunoprecipitated (IP) and immunoblotted. (**e**) Non-phosphorylated parafibromin specifically interacts with NICD. HEK293T cells were transfected with the indicated vectors. TCLs were immunoprecipitated with the indicated antibodies and immunoprecipitates (IP) were incubated with recombinant phosphorylated/non-phosphorylated parafibromin. The reaction mixtures were subjected to immunoblotting. (**f**) PR-parafibromin shows increased ability to activate Notch signalling. Notch-responsive luciferase reporter assays were performed in HEK293T cells (*n*=3, mean±s.d. ^**^P<0.01; ANOVA with Bonferroni's *post hoc* test). (**g**) MCF7 cells were transiently transfected with a parafibromin expression vector. At 48 h after transfection, TCLs were subjected to immunoblotting. (**h**) Parafibromin inhibits proteasome-dependent degradation of NICD. HEK293T cells were transfected with the indicated vectors and incubated with or without 10 μM MG132 for 8 h. TCLs were immunoblotted with the indicated antibodies. (**i**) Expression of PR-parafibromin induces transactivation of Notch target genes. MCF7 cells were transfected with the indicated vectors and mRNA expression levels of *HEY1* and *HES1* were measured by RT–qPCR analyses (*n*=4, mean±s.d. *P<0.05, ^**^P<0.01; a two-tailed unpaired Student's *t*-test). (**j**) Conditional deletion of parafibromin in *Hrpt2*^*flox/flox*^ MEFs was induced by 4-OHT treatment and expression levels of *Hey1* and *Hes1* were measured by RT–qPCR analyses (*n*=3, mean±s.d. ^**^P<0.01; a two-tailed unpaired Student's *t*-test). Parafibromin knockout was confirmed by immunoblotting.

In zebrafish, Rtf1 and Ctr9, components of the PAF complex, are required for the activation of Notch signalling during embryogenesis^25^. However, the role of parafibromin, a core component of the PAF complex, in the regulation of Notch signalling has remained unclear. To address this issue, we performed a Notch-dependent luciferase reporter assay in HEK293T cells and found that knockdown of parafibromin significantly reduced the reporter activity and that this reduction was rescued by ectopic expression of RNAi-resistant parafibromin (Fig. 2a). The result of the experiment suggested that parafibromin potentiated Notch signalling. Given that parafibromin enhances both Wnt signalling and Hh signalling by interacting with their respective effectors, β-catenin and Gli1, we hypothesized that parafibromin also potentiates Notch signalling by interacting with the Notch effector, the NICD. To test this idea, we carried out a co-immunoprecipitation study using an anti-parafibromin antibody in human MCF7 breast cancer cells, in which Notch signalling is constitutively activated^26^. We found that NICD was co-immunoprecipitated with endogenous parafibromin (Fig. 2b and Supplementary Fig. 2a,b), indicating that parafibromin formed a complex with NICD in the cell. Furthermore, treatment of HEK293T cells and MCF7 cells with a γ-secretase inhibitor, DAPT, abrogated parafibromin-dependent Notch reporter activation as well as parafibromin/NICD complex formation (Fig. 2c and Supplementary Fig. 2c), indicating that potentiation of the Notch signal by parafibromin requires γ-secretase-dependent release of NICD. To know if the phosphorylation status of parafibromin affects the complex formation, we transiently expressed wild-type or PR-parafibromin together with NICD in HEK293T cells and we found that PR-parafibromin co-immunoprecipitated a greater amount of NICD than wild-type parafibromin did (Fig. 2d and Supplementary Fig. 2d). Furthermore, *in vitro* binding assay using recombinant parafibromin revealed that non-phosphorylated parafibromin, but not tyrosine-phosphorylated parafibromin, bound to NICD (Fig. 2e). Thus, parafibromin formed a complex with NICD in a manner that was dependent on tyrosine dephosphorylation of parafibromin. Consistent with this observation, PR-parafibromin was more potent in activating Notch signalling than wild-type parafibromin was in the Notch reporter assay (Fig. 2f). Treatment of cells with DAPT reduced endogenous Notch signalling, whereas NICD-induced activation of the Notch reporter was little influenced by treatment with DAPT (Supplementary Fig. 2e).

Also notably, ectopic expression of parafibromin elevated the amount of ectopically co-expressed NICD (Fig. 2d,f). Likewise, the level of endogenous NICD was elevated on parafibromin expression in MCF7 cells (Fig. 2g). However, the proteasome inhibitor MG132 failed to further increase the level of NICD, which had already been elevated by parafibromin (Fig. 2h), suggesting that parafibromin stabilized NICD by inhibiting proteasome-dependent degradation. In the presence of MG132, ectopic expression of parafibromin was still capable of potentiating Notch signal activation (Supplementary Fig. 2f). These results collectively indicated that parafibromin potentiated Notch signalling both quantitatively (via NICD stabilization) and qualitatively (as a transcriptional coactivator). In fact, ectopic expression of PR-parafibromin in MCF7 cells gave rise to elevated mRNA and protein levels of Notch-target genes *HES1* and *HEY1*, which were blocked by DAPT treatment (Fig. 2i and Supplementary Fig. 2g,h). Conversely, conditional deletion of the floxed *Hrpt2* alleles in mouse embryonic fibroblasts (MEFs) resulted in reduced expression of *Hey1* and *Hes1* (Fig. 2j). Thus, as are the cases with the Wnt and Hh signalling pathways, parafibromin functioned as a transcriptional coactivator in the Notch signalling pathway by forming a physical complex with NICD.

### Parafibromin integrates the Wnt and Hedgehog signals

**Figure 3:**
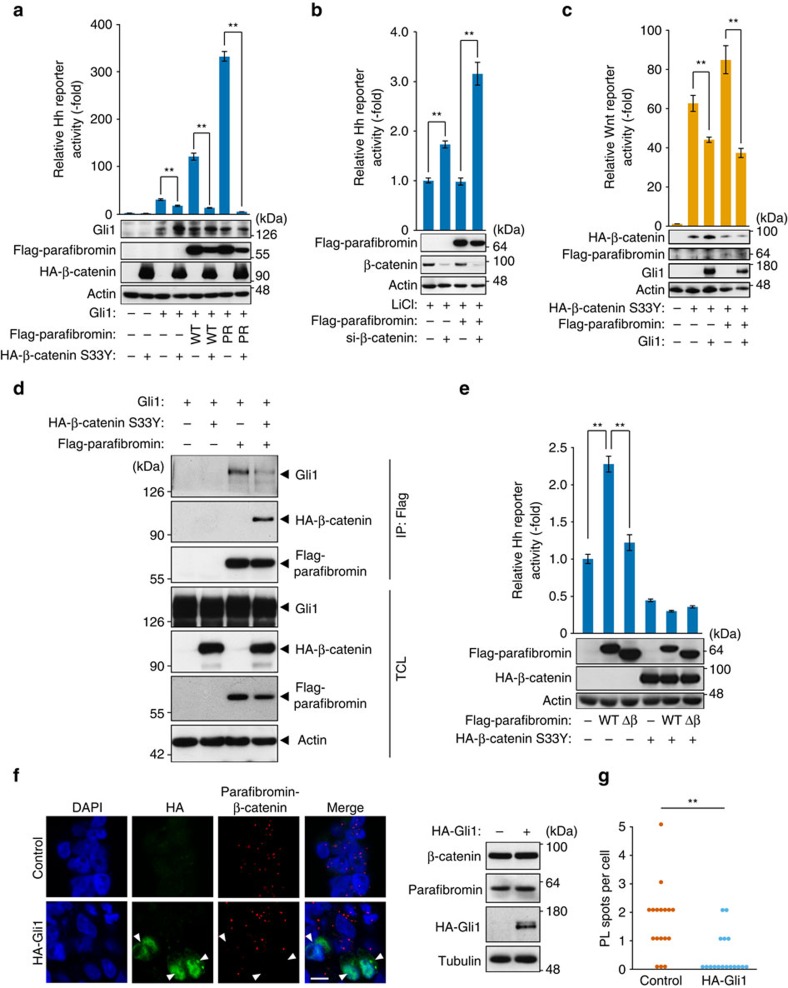
Parafibromin activates Wnt and Hedgehog signals in a mutually exclusive manner. (**a**,**b**) β-catenin competitively inhibits parafibromin-dependent Hh signalling. Hh-responsive luciferase reporter assays were performed in HEK293T cells. Total cell lysates (TCLs) were subjected to immunoblotting (*n*=3, mean±s.d. ^**^P<0.01; ANOVA with Bonferroni's *post hoc* test). For **b**, transfected cells were treated with 25 mM LiCl for 24 h. (**c**) Gli1 suppresses parafibromin-dependent Wnt signalling. Wnt-responsive luciferase reporter assays were performed in HEK293T cells (*n*=3, mean±s.d. ^**^P<0.01; ANOVA with Bonferroni's *post hoc* test). (**d**) β-catenin competitively inhibits parafibromin/Gli1 interaction. HEK293T cells were transfected with the indicated vectors. TCLs were sequentially immunoprecipitated (IP) and immunoblotted. (**e**) A parafibromin mutant lacking β-catenin-binding site (Δβ) failed to potentiate Hh signal. Hh-responsive luciferase reporter assays were performed in HEK293T cells transfected with the Gli1 expression vector. Total cell lysates (TCLs) were subjected to immunoblotting (*n*=3, mean±s.d. ^**^P<0.01; ANOVA with Bonferroni's *post hoc* test). (**f**) Proximity ligation assay (PLA) in HEK293T cells. Transfected cells were treated with 25 mM LiCl. Red spots indicate the parafibromin/β-catenin interaction. Nuclei and Gli1-expressing cells were stained in blue (DAPI) and green, respectively. Scale bar, 10 μm. Protein expression levels in control and Gli1-expressing cells were indicated by immunoblotting. (**g**) Data for quantitative analysis of the PL spots; *n*=17. ^**^P<0.01; Wilcoxon rank sum test.

The above-described observations demonstrated that parafibromin acted as a common transcriptional coactivator in the Wnt, Hh and Notch signalling pathways, thereby promoting downstream target gene activation. This led us to pursue the possibility that parafibromin serves as a molecular platform that integrates multiple morphogen signals and converts them to proper transcriptional outputs. To test this idea, we examined the effect of Wnt signal activation on the parafibromin-dependent Hh signal potentiation. Inhibition of parafibromin expression by shRNA decreased both endogenous Wnt and Hh signals in HEK293T cells (Supplementary Fig. 3a). Furthermore, knockdown of β-catenin by specific siRNA gave rise to the potentiation of Hh signalling (Supplementary Fig. 3b). These observations indicated that parafibromin was utilized for the reciprocal activation of the Wnt and Hh signalling pathways. Consistent with these, co-expression of a constitutively active form of β-catenin (β-catenin S33Y) markedly suppressed Hh signalling activated by parafibromin and Gli1 in HEK293T cells (Fig. 3a). Likewise, LiCl treatment of cells, which inhibits GSK3β and thereby prevents β-catenin degradation, also inhibited parafibromin-dependent Hh activation (Supplementary Fig. 3c). Conversely, knockdown of β-catenin in LiCl-treated cells restored parafibromin-dependent activation of Hh signalling (Fig. 3b). In a reciprocal experiment, ectopic expression of Gli1 attenuated Wnt activation by parafibromin (Fig. 3c). Accordingly, parafibromin-dependent Wnt activation inhibited parafibromin-dependent Hh activation and vice versa. To examine mutual inhibition between the Wnt signal and Hh signal in more physiological settings, we made use of MKN28 human gastric epithelial cells. In MKN28 cells, a Wnt3a/R-spondin-conditioned medium induced nuclear accumulation of β-catenin and activated the Wnt reporter (Supplementary Fig. 3d,e), whereas expression of the Hh ligand, Sonic hedgehog (Shh), stimulated the Hh signal (Supplementary Fig. 3f,g). Thus, MKN28 cells respond to both Wnt and Hh ligands. The Hh signal activation by Shh in MKN28 cells was substantially attenuated when the cells were co-stimulated with the Wnt3a/R-spondin-conditioned medium (Supplementary Fig. 3h).

Since the proposed β-catenin-binding site (aa 218–263) and Gli1-binding site (aa 200–343) on parafibromin are overlapping^20^,^23^, we assumed that the observed reciprocal inhibition between the Wnt signal and the Hh signal was due to competition between β-catenin and Gli1 for parafibromin binding. To test this idea, we performed a co-immunoprecipitation study in HEK293T cells and found that parafibromin/Gli1 interaction was substantially reduced on co-expression of β-catenin, which formed a complex with parafibromin (Fig. 3d). Wnt signal activation by LiCl treatment also reduced parafibromin/Gli1 interaction (Supplementary Fig. 3i). These results indicated that β-catenin competitively inhibits parafibromin/Gli1 interaction, thereby diminishing Hh signal activation. Consistent with this, we observed that a parafibromin mutant lacking the β-catenin-binding site (aa 218–263) failed to potentiate the activation of the Hh signal by Gli1 (Fig. 3e), suggesting that the two pathways require the same parafibromin region for signal activation.

Next, to test if Gli1 competitively inhibits parafibromin/β-catenin interaction, we ectopically expressed Gli1 in LiCl-treated HEK293T cells and quantitatively measured parafibromin/β-catenin complex formation in cells by an *in situ* proximity ligation assay (PLA). The result of the experiment revealed that Gli1-expressing cells exhibited a significant reduction in parafibromin/β-catenin interaction (Fig. 3f,g). Taken together, these results indicated that reciprocal inhibition between the Wnt signal and Hh signal was due to competition between β-catenin and Gli1 for parafibromin binding.

### Parafibromin integrates the Wnt and Notch signals

**Figure 4:**
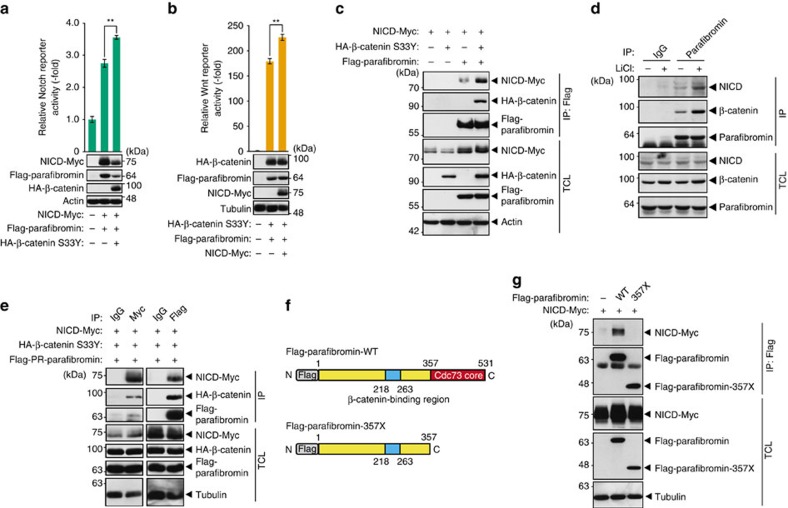
Parafibromin activates Wnt and Notch signals in a cooperative manner. (**a**,**b**) Wnt and Notch signals mutually potentiate parafibromin-dependent signal activation. Luciferase reporter assays using the indicated reporter plasmids in HEK293T cells. Total cell lysates (TCLs) were subjected to immunoblotting (*n*=3, mean±s.d. ^**^P<0.01; ANOVA with Bonferroni's *post hoc* test). (**c**) β-catenin stabilizes parafibromin/NICD interaction. HEK293T cells were transfected with the indicated vectors. TCLs were sequentially immunoprecipitated (IP) and immunoblotted with the indicated antibodies. (**d**) Endogenous interaction between parafibromin, NICD and β-catenin was increased on endogenous Wnt signal activation. HEK293T cells treated with or without 25 mM LiCl were lysed and sequentially immunoprecipitated (IP) with an anti-parafibromin antibody and immunoblotted. (**e**) Parafibromin forms a trimeric complex with β-catenin and NICD. HEK293T cells were transfected with the indicated vectors. TCLs were sequentially immunoprecipitated (IP) with the indicated antibodies and immunoblotted. (**f**) A schematic view of Flag-tagged wild-type parafibromin and its derivative, parafibromin-357X. The cdc73 core homology domain (aa 357–531) and β-catenin-binding site (aa 218–263) are indicated. (**g**) The C-terminal domain of parafibromin is required for NICD binding. HEK293T cells were transfected with the indicated vectors. TCLs were sequentially immunoprecipitated (IP) with the indicated antibodies.

In the mammalian intestinal epithelia, maintenance of intestinal stem cells and proliferative progenitor cells requires the cooperative activation of Wnt and Notch signalling^10^,^11^. However, molecular mechanisms underlying cooperative activation of these two pathways remain poorly understood. To examine the effect of Wnt signal activation on parafibromin-dependent Notch signal activation, we co-expressed parafibromin, NICD and β-catenin in HEK293T cells and performed a Notch-dependent reporter assay. In contrast to the case of Hh signalling, β-catenin enhanced rather than inhibited parafibromin-dependent Notch activation (Fig. 4a). We also found that endogenous Wnt signal activation by LiCl promoted endogenous Notch signalling in HEK293T cells (Supplementary Fig. 4a). Reciprocally, NICD potentiated parafibromin-dependent Wnt activation (Fig. 4b). Thus, parafibromin stimulated Wnt- and Notch-dependent gene activation in a cooperative manner. To gain insights into the mechanism underlying the signal cooperation, we examined the effect of β-catenin on parafibromin/NICD interaction and we found that parafibromin/NICD interaction was strengthened in the presence of ectopic β-catenin (Fig. 4c). We also examined endogenous protein interaction using HEK293T cells and found that endogenous NICD and endogenous β-catenin were co-precipitated with endogenous parafibromin (Fig. 4d). Furthermore, the complex formation between these endogenous proteins was substantially potentiated upon stimulation of Wnt signalling by LiCl (Fig. 4d). These results indicated that β-catenin stimulated parafibromin/NICD complex formation and thereby promoted parafibromin-dependent Notch signal activation. The notion that β-catenin did not compete with parafibromin/NICD interaction raised the possibility that β-catenin and NICD simultaneously interact with parafibromin and thereby form a heterotrimeric complex. To test this idea, we triply expressed parafibromin, β-catenin and NICD in HEK293T cells and performed a co-immunoprecipitation study. Immunoprecipitation of parafibromin or NICD from lysates prepared from the triple-transfected HEK293T cells co-immunoprecipitated the other two proteins (Fig. 4e), indicating that the three proteins are present in a complex in the cell. This idea was further supported by the data showing that the C-terminal Cdc73 core homology domain of parafibromin (aa 357–531), which is located separate from the N-terminal β-catenin-binding site (aa 218–263), was required for NICD binding as well as potentiation of Notch signalling (Fig. 4f,g and Supplementary Fig. 4b). On the basis of these observations, we concluded that parafibromin co-stimulated the Wnt and Notch signals by forming a complex with β-catenin and NICD, respectively.

### PTK6 impairs the coactivator function of parafibromin

**Figure 5:**
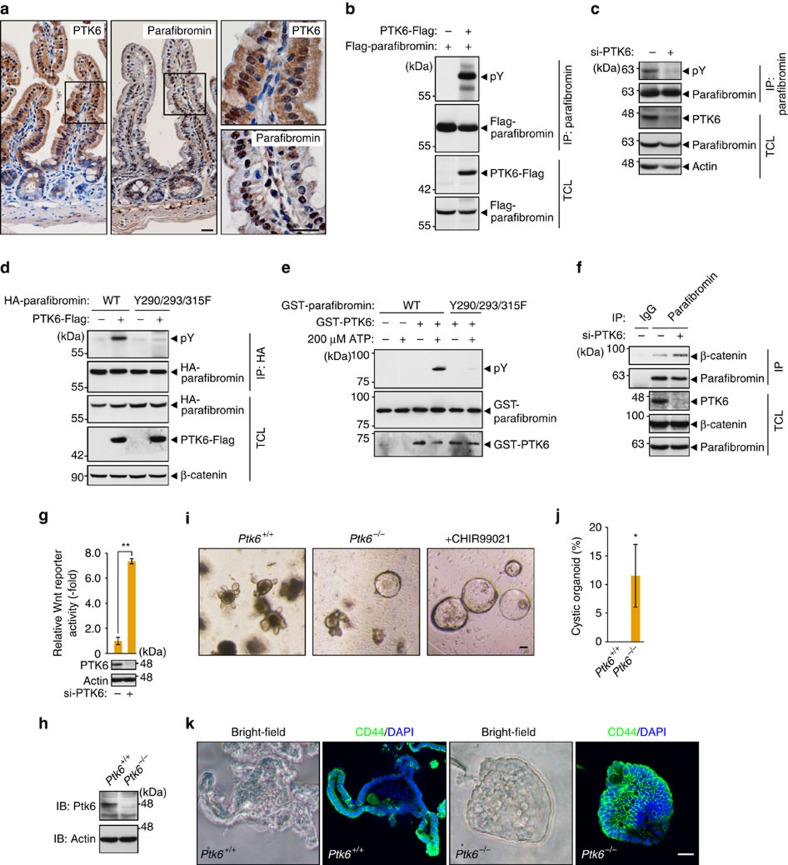
PTK6 phosphorylates parafibromin and impairs its scaffold function. (**a**) Immunohistochemistry of the mouse small intestine confirming the co-expression of PTK6 and parafibromin. The right panels show higher-magnification images. Scale bars, 20 μm. (**b**,**c**) PTK6 regulates parafibromin tyrosine phosphorylation. AGS cells were transfected with the indicated vectors (**b**) or si-PTK6 (**c**). Total cell lysates (TCLs) were sequentially immunoprecipitated (IP) with the indicated antibodies. (**d**) Y290/293/315F mutations abolish parafibromin phosphorylation by PTK6. AGS cells were transfected with the indicated vectors. TCLs were sequentially immunoprecipitated (IP) with the indicated antibodies and immunoblotted. (**e**) *In vitro* kinase assays indicating parafibromin is a direct substrate of PTK6. Purified recombinant parafibromin and PTK6 were incubated with 200 μM ATP. The reaction mixtures were subjected to immunoblotting. (**f**) PTK6 regulates parafibromin/β-catenin interaction. AGS cells were transfected with si-PTK6. TCLs were sequentially immunoprecipitated (IP) with the indicated antibodies. (**g**) PTK6 knockdown in AGS cells resulted in increased Wnt-reporter activity in the luciferase reporter assay (*n*=3, mean±s.d. ^**^P<0.01; a two-tailed unpaired Student's *t*-test). (**h**–**k**) *Ptk6*^*−/−*^ intestinal organoids show less-differentiated ‘cystic' growth and expanded Wnt signal activation. Immunoblot analysis (**h**), morphology (**i**), a percentage of cystic organoids (**j**) and CD44 immunostaining (**k**) of control and *Ptk6*^*−/−*^intestinal organoids (*n*=3, mean±s.d. *P<0.05; Wilcoxon rank sum test). Scale bar, 50 μm.

Whereas SHP2 has been shown to mediate dephosphorylation of parafibromin^21^, little is known about a kinase(s) that inhibits the platform function of parafibromin through tyrosine phosphorylation. To address this issue, we focused on PTK6, also known as BRK, an intracellular tyrosine kinase distinctly related to Src family kinases^27^. Physiologically, PTK6 is primarily expressed in differentiated non-dividing epithelial cells of the gastrointestinal tract^28^. We observed that both PTK6 and parafibromin were expressed in the nucleus of differentiated epithelial cells above the crypt–villus border of the mouse small intestine (Fig. 5a). To test if PTK6 phosphorylates parafibromin, we co-expressed PTK6 and parafibromin in AGS human gastric cancer cells. We found that PTK6 expression markedly elevated the level of tyrosine phosphorylation of parafibromin (Fig. 5b). Conversely, knockdown of PTK6 in AGS cells resulted in reduced levels of parafibromin tyrosine phosphorylation (Fig. 5c and Supplementary Fig. 5a). Furthermore, PR-parafibromin (Y290/293/315F) was totally resistant to tyrosine phosphorylation by PTK6 (Fig. 5d). An *in vitro* kinase assay consolidated direct phosphorylation of parafibromin on Y290/293/315 (Fig. 5e). These observations collectively indicated that PTK6 is the kinase that mediates parafibromin phosphorylation at Y290/293/315, which are reciprocally dephosphorylated by SHP2 (ref. 21). To study the functional consequence of parafibromin phosphorylation by PTK6, we examined the effect of PTK6 inhibition on Wnt signal activation. Knockdown of PTK6 in AGS cells enhanced endogenous parafibromin/β-catenin complex formation (Fig. 5f) and potentiated Wnt-reporter activity (Fig. 5g). Thus, PTK6 actively suppressed the Wnt signalling pathway in AGS cells through tyrosine phosphorylation of parafibromin.

To further investigate the physiological role of PTK6, we made *Ptk6* knockout mice by using clustered regulatory interspaced short palindromic repeats (CRISPR)/Cas-mediated genome editing^29^,^30^ (Supplementary Fig. 5b). Immunohistochemistry and immunoblot analysis using an anti-Ptk6 antibody confirmed the loss of Ptk6 expression in *Ptk6*^*−/−*^ mice (Fig. 5h and Supplementary Fig. 5c). We then performed intestinal organoid culture^31^ using crypts isolated from *Ptk6*^*−/−*^ mice and its wild-type littermates. We found that *Ptk6*^*−/−*^ organoids exhibited less-differentiated ‘cystic' growth^32^ resembling the effect of Wnt activation by GSK3β inhibitor CHIR99021 (Fig. 5i,j). Consistent with this, *Ptk6*^*−/−*^ organoids showed expanded expression pattern of the Wnt target CD44 compared with wild-type organoids (Fig. 5k). These observations indicated the importance of Ptk6 in counteracting Wnt signalling in the maintenance of intestinal homeostasis.

### Loss of parafibromin disorganizes the intestinal epithelium

**Figure 6:**
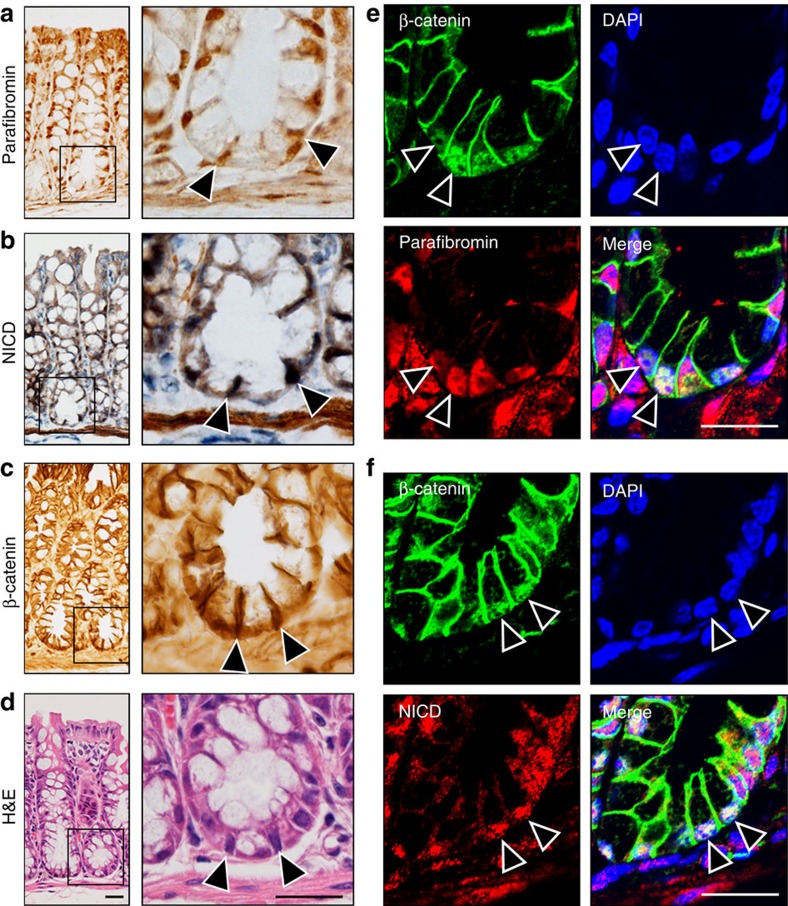
Co-expression of proteins in intestinal crypts. (**a**–**d**) Parafibromin (**a**), NICD (**b**) and β-catenin (**c**) immunohistochemistry of the mouse colon. H&E staining of the serially sectioned sample is shown in **d**. The right panels show higher-magnification images of crypt bases. Arrowheads indicate the positive nuclear staining of each antibody. Scale bars, 20 μm. (**e**,**f**) Immunohistochemical analysis of β-catenin and parafibromin expression (**e**) and β-catenin and NICD expression (**f**) in the bottom region of colonic crypts. Nuclei were stained in blue (DAPI). Arrowheads indicate the cells showing co-localization of parafibromin, β-catenin and NICD. Scale bars, 20 μm.

**Figure 7:**
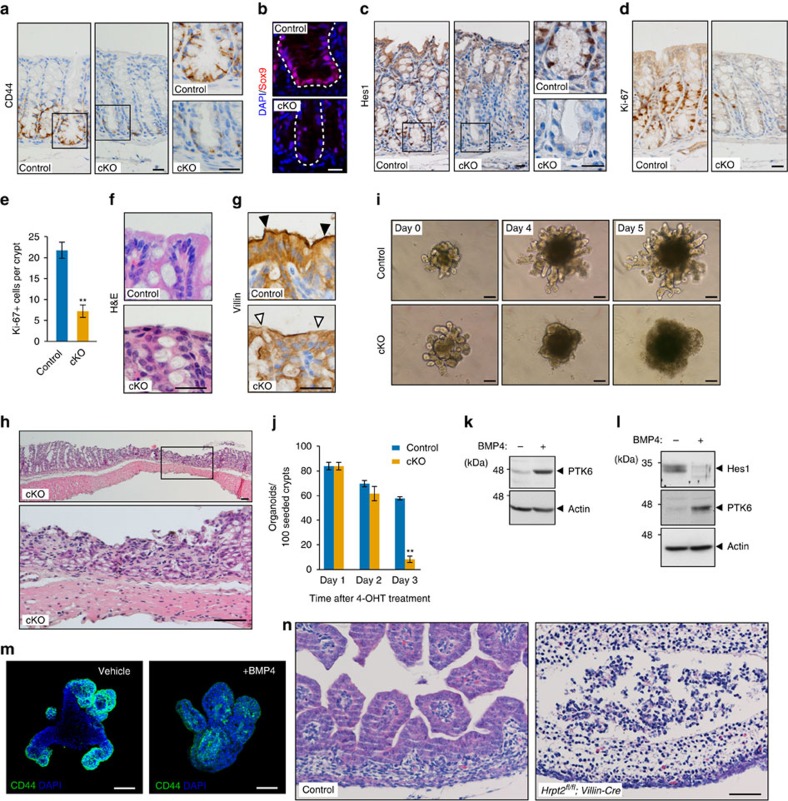
Loss of parafibromin leads to disorganization of the intestinal epithelium. (**a**–**c**) Conditional knockout (cKO) of *parafibromin* results in decreased expression of Wnt and Notch targets in the intestine. CD44 immunohistochemistry (**a**), Sox9 immunofluorescent staining (**b**) and Hes1 immunohistochemistry (**c**) of control and *parafibromin* cKO colons. The right panels show higher-magnification images. Scale bars, 20 μm. (**d**,**e**) Parafibromin is essential for the intestinal epithelial cell proliferation. Ki-67 immunohistochemistry (**d**) and number of Ki-67-positive cells per crypt (**e**) (*n*=4 animals per group. Means±s.d.; ^**^P<0.01; a two-tailed unpaired Student's *t*-test). Scale bar, 20 μm. (**f**,**g**) Effect of *parafibromin* cKO on enterocyte differentiation. Haematoxylin and eosin (H&E) staining (**f**), and Villin immunohistochemistry (**g**) of colonic epithelial tissues from control and *parafibromin* cKO mice. (**h**) Parafibromin is essential for the maintenance of intestinal epithelium. H&E staining was performed in colon sections from *parafibromin* cKO mice. Scale bars, 50 μm. (**i**,**j**) *Parafibromin* cKO results in demise of intestinal organoids. Time-lapse image (**i**) and number (**j**) of intestinal organoids established from control and *parafibromin* cKO mice at each day after 4-OHT treatment (*n*=3, mean±s.d. ^**^P<0.01; a two-tailed unpaired Student's *t*-test). Scale bars, 100 μm. (**k**–**m**) BMP signalling regulates PTK6 expression. Intestinal organoids were treated with recombinant BMP4 and subjected to immunoblotting (**k**,**l**) or CD44 immunostaining (**m**). (**n**) Parafibromin is essential for the development of the intestinal epithelium. H&E staining of embryonic intestinal tissues from control and *Hrpt2*^*flox/flox*^/*Villin-Cre* embryos at E16.5. Scale bars, 50 μm.

To determine the *in vivo* relevance of parafibromin-mediated morphogen signal coordination, we analysed the expression of parafibromin and Wnt/Notch-related molecules in the mouse intestinal epithelium. We found that the Wnt targets CD44 and Sox9 and the Notch target Hes1 were expressed in cells at the bottom region of colonic crypts (Supplementary Fig. 6a–c), confirming that Wnt and Notch signals are simultaneously activated in this region where parafibromin, NICD and β-catenin were all expressed (Fig. 6a–f). To further elucidate *in vivo* roles of parafibromin, we used *parafibromin* conditional knockout (cKO) mice (*Hrpt2*^*flox/flox*^*/CAG-CreER* mice)^33^. On acute deletion of *parafibromin* (Supplementary Fig. 7a), the intestinal epithelium exhibited a marked reduction in expression of the Wnt targets CD44 and Sox9 (Fig. 7a,b) and the Notch target Hes1 (Fig. 7c). Thus, parafibromin was indispensable for activation of the Wnt and Notch signals in the adult intestinal epithelium. Furthermore, *parafibromin* cKO crypts exhibited significant reduction in the number of Ki-67-positive cells (Fig. 7d,e), indicating that parafibromin was indispensable for crypt cell proliferation, which depends on coordinated Wnt and Notch activation. Since *parafibromin* cKO crypts did not contain TUNEL-positive cells and retained normal expression of Gapdh and SHP2 proteins (Supplementary Fig. 7b,c), the observed changes in cKO crypts were not due to apoptosis. In control mice, differentiated enterocytes were present at the top of colonic crypts (Fig. 7f,g). In contrast, the *parafibromin* cKO colonic mucosa lost columnar morphology of enterocytes with defective microvilli formation (Fig. 7f,g). Since Hh signal activation in sub-villus mesenchymal cells is essential for enterocyte differentiation^7^, these abnormalities might be due to impaired Hh signalling caused by the loss of parafibromin. Consequently, the intestinal mucosa underwent severe structural disintegration even at 5 days after tamoxifen treatment (Fig. 7h). We also performed intestinal organoid culture using intestinal crypts isolated from *parafibromin* cKO mice and found that conditional removal of parafibromin results in the demise of intestinal organoids (Fig. 7i,j). Using intestinal organoids, we also investigated the upstream signal regulating the expression of PTK6. In the intestinal epithelium, BMP secreted from villous mesenchymal cells induces epithelial cell differentiation^34^. Accordingly, we assumed that BMP induces PTK6 in intestinal epithelial cells and thereby represses parafibromin-mediated activation of the Wnt and Notch signals in maturing epithelial cells. To test this possibility, we treated intestinal organoids with BMP4. We found that BMP4 stimulation induced PTK6 expression (Fig. 7k), which was concomitantly associated with reduced levels of the Notch target Hes1 and Wnt target CD44 (Fig. 7l,m). These observations indicated that BMP-induced PTK6 inhibited the ability of parafibromin to stimulate Wnt and Notch signals by tyrosine phosphorylation. We also generated *Hrpt2*^*flox/flox*^/*Villin-Cre* mice in which intestinal epithelium-specific Cre-dependent recombination is initiated at embryonic day 9 (E9). Strikingly, *Hrpt2*^*flox/flox*^/*Villin-Cre* mouse embryos failed to develop the embryonic intestinal epithelium, which was formed in wild-type littermates by E16.5 (Fig. 7n). These observations provided compelling evidence for the indispensable role of parafibromin in the development and maintenance of intestinal epithelial linings.

## Discussion

**Figure 8:**
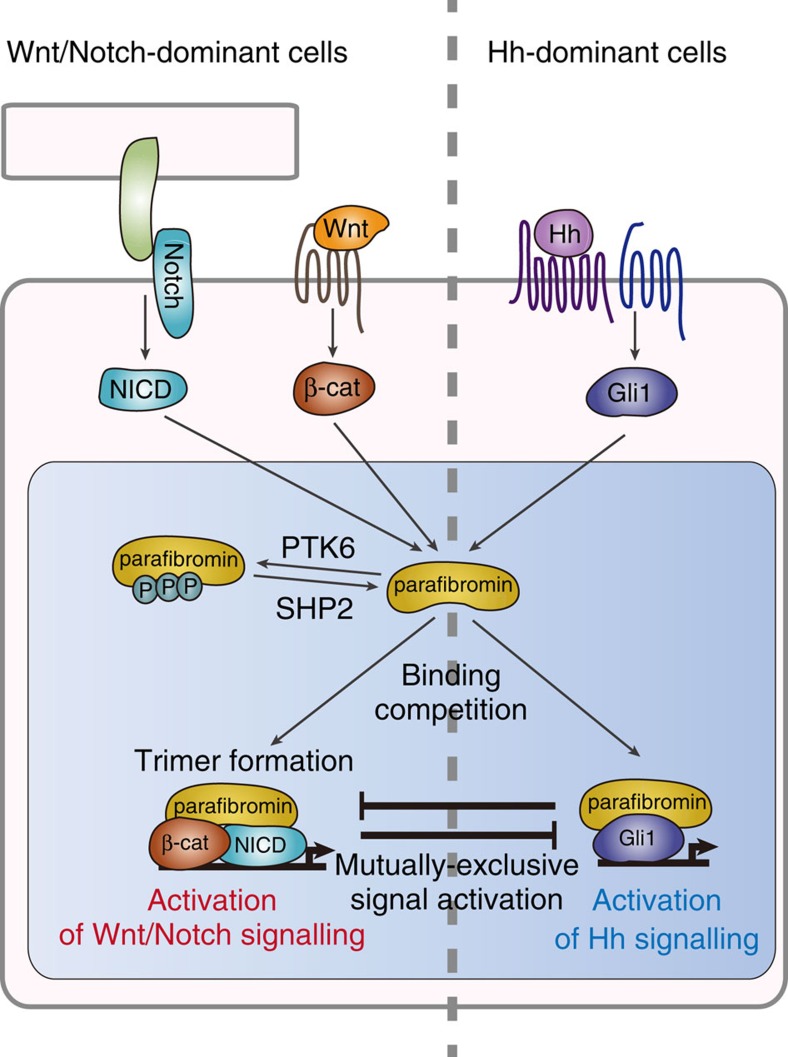
A schematic diagram showing parafibromin-mediated signal coordination. Signal inputs and outputs of the Wnt, Hh and Notch signals are coordinated intracellularly by parafibromin, which acts as a transcriptional platform/scaffold. Depending on its cellular context, parafibromin competitively interacts with β-catenin or Gli1, thereby activating Wnt and Hh signals in a mutually exclusive manner. On the other hand, parafibromin forms a heterotrimeric complex with β-catenin and Notch intracellular domain (NICD), supporting concerted activation of Wnt and Notch signals. This platform function of parafibromin is inversely regulated by parafibromin tyrosine phosphorylation and dephosphorylation, which are mediated by PTK6 and SHP2, respectively.

Morphogens such as Wnt, Hedgehog and Notch are protein ligands that are present as secreted or membrane-bound forms, and they create concentration gradients to provide positional cues for organ development and tissue homeostasis^1^,^2^,^3^. In many cases, the Wnt and Notch signalling pathways play important roles in cell fate decision-making of the stem/progenitor cell compartment^35^, whereas Hh signalling transmits information to progenitor cells required for proper differentiation in both development and homeostasis^36^. At a single-cell level, these morphogen signals activate parallel information-processing conduits that converge onto the transcriptional effectors, which govern expression of genes specific to each morphogen. An important question here is how signals independently triggered by distinct morphogens are integrated and shaped intracellularly so that they can generate appropriate cellular responses in a spatiotemporally regulated manner. The present study revealed that, in addition to ligand production/receptor expression and cross-regulation/cross-talk at the levels of cytoplasmic signal transducers, a cellular response to these morphogens is determined by a combination of signal effectors aligned on parafibromin, which acts as a nuclear platform that integrates multiple distinct morphogen signals and converts them to integrated transcriptional outputs (a schematic diagram is depicted in Fig. 8).

Mechanistically, parafibromin interacts with the Wnt effector β-catenin, the Hh effector Gli1, and the Notch effector NICD and thereby potentiates their transcriptional effector functions. Furthermore, parafibromin competitively binds to β-catenin and Gli1 via the N-terminal metazoan-specific region, indicating reciprocal activation of canonical Wnt signalling and Hh signalling by parafibromin like ‘musical chairs'. Such a situation may physiologically occur in normal tongue epithelial cells^37^ as well as the hair bud cells^38^, which react both to Wnt and Hh ligands and, consistent with our present findings, Wnt activation suppresses Hh signalling and vice versa. Reciprocal activation of Wnt and Hh signalling has also been suggested in the neural tube along the dorso-ventral axis^8^,^9^ and in intestinal epithelia along the crypt–villus axis^7^. In the intestine, however, the Hh receptor Patched and Gli are expressed exclusively on myofibroblasts in the villous stroma^39^, arguing against the notion of intracellular competition between Wnt and Hh signals in epithelial cells. Nevertheless, the results of the present study indicate a molecular mechanism through which intestinal epithelial cells acquire unresponsiveness to Hh ligands. In the crypt, strong Wnt stimulation leads to complex formation of parafibromin exclusively with β-catenin (first come, first served) in epithelial cells. In the absence of the parafibromin–Gli1 complex, expression of PTCH and Gli1 is not allowed in stem/progenitor cells and descendant cells including villus epithelial cells, eventually leading to fixation of cellular responsiveness in terms of morphogen stimuli at a single-cell level. In human colorectal cancers, in which Wnt signalling is aberrantly deregulated, cancer cells show an inverse correlation in nuclear staining of β-catenin and Gli1^40^. Mutually exclusive activation of the Wnt and Hh pathways has also been observed in human gastric cancer cells^41^. Since neoplastic transformation is characterized by dedifferentiation or acquisition of stemness^42^, the dual responsiveness of gastrointestinal cancer cells to Wnt and Hh ligands may reflect their stemness-related property. In contrast to the case of β-catenin and Gli1, binding of parafibromin with the Notch signal effector NICD requires the C-terminal region that is conserved in yeast Cdc73. Consequently, parafibromin can simultaneously interact with β-catenin and NICD and thus promote their transcriptional activities. The finding is consistent with the observation that both Wnt and Notch activities are required for maintenance of the intestinal crypt compartment^10^,^11^. Also, deregulated Notch signalling functionally collaborates with deregulated Wnt activation in intestinal tumorigenesis^10^,^11^, supporting the idea that the two pathways may cooperate in stimulating proliferation of intestinal stem and/or progenitor cells in the crypt.

The results of the present study revealed an additional layer of morphogen signal regulation by parafibromin. Parafibromin undergoes tyrosine-phosphorylation and -dephosphorylation on Y290/293/315 by PTK6 and SHP2 (ref. 21), respectively. The ability of parafibromin to interact with β-catenin or Gli1 requires tyrosine dephosphorylation on these residues. Since these tyrosine residues are located in close proximity to the binding region of β-catenin or Gli1 (residues 218–263), phosphorylation may provoke a steric change of parafibromin that disables interaction with β-catenin or Gli1. Notably, interaction of parafibromin with NICD, which does not compete with β-catenin or Gli1, is also regulated by parafibromin dephosphorylation. It is therefore possible that the structure of the target protein-binding surface of parafibromin is extensively altered by the status of tyrosine phosphorylation/dephosphorylation. PTK6, also known as BRK, is a tyrosine kinase that is highly expressed in non-dividing villous epithelial cells in the intestinal tract^43^. PTK6 is induced in intestinal organoids by treatment with BMP4. Involvement of PTK6 in intestinal homeostasis was demonstrated by the observation that organoid formation, especially formation of the villus component, was impaired when organoids were prepared from *Ptk6* knockout mice. The results therefore indicate that parafibromin is the transcriptional scaffold working primarily in the crypt component. *Ptk6* knockout mice exhibit excess proliferation of crypt cells and an increase in the number of villus epithelial cells with delayed differentiation^44^. Conversely, inhibition of BMP signalling potentiates robustness of intestinal cell stemness by enhancing the Wnt signal, resulting in the formation of numerous ectopic crypt units^34^. The inhibitory role of PTK6 in Wnt signalling raises the idea that the kinase may be downregulated in colorectal carcinogenesis. Indeed, a recent study showed that the level of PTK6 expression was decreased in clinically isolated human colorectal cancers^45^.

Acute systemic loss of parafibromin in adult mice gave rise to disorganization of the intestinal epithelial architecture. These mice died within 20 days after being exposed to tamoxifen^33^. Before death, the mice showed low activity, laboured breathing, slow reaction, weight loss, and a reduction in abdominal and subcutaneous adipose tissue, which was concomitantly associated with rapid onset of cachexia. Some of the mice also developed dilation of the gastrointestinal tract and ascites formation. Whereas the observed phenotype was overwhelming and made it difficult to identify a primary lesion that underlies the pathological lesions, the striking phenotype is due most likely to the abolition of the alimentary tract, which is consistent with the role of parafibromin on morphogen signals proposed in the present study (Fig. 8). However, given that parafibromin is also involved in the multifunctional PAF complex^17^,^18^,^19^, the devastating intestinal lesions induced by loss of parafibromin may not be fully explained by attenuation of morphogen signalling. A critical role of parafibromin in the development and maintenance of the intestinal tract has also been supported by the results of the present study showing that acute loss of parafibromin impaired intestinal organoid formation/maintenance.

Although there remains the possibility that the effect of parafibromin on morphogen-regulated transcription simply reflects a more general role of parafibromin as a component of the PAF complex^17^,^18^,^19^, which has been linked to diverse steps of transcription-related processes, we consider that this possibility is unlikely because parafibromin was still capable of efficiently stimulating the Wnt signal in cells in which formation of the PAF complex was inhibited by knocking down PAF1 (ref. 21). Also notably, only a very few genes are directly regulated by the PAF complex in yeast^16^,^46^. Acute loss of parafibromin in MEF cells resulted in downregulation of only 68 transcripts out of 15,300 transcripts^33^, which included Wnt-target genes such as *Hmga1* and *Hmga2* (refs 47, 48) and Notch target genes such as *Igf2* and *H19* (ref. 49). In Arabidopsis, the effect of parafibromin inactivation is primarily limited to flowering time, whereas other PAF component mutants exhibit pleiotropic developmental phenotypes^50^. These observations argue against the idea that the PAF complex is broadly involved in transcription as a component of the basal transcription machinery while suggesting that parafibromin has its own unique role independent of the PAF complex or that it is required for the function of the PAF complex at only a small subset of target genes.

The present work revealed that multiple independent morphogen signalling pathways converge on the transcriptional platform parafibromin. As far as we know, the retinoblastoma (pRb) protein is the only molecule having similarity with parafibromin in that it controls a number of transcription factors by complex formation, while the scaffold function is regulated by phosphorylation^51^. Interestingly, both parafibromin and pRB function as tumour suppressors. Given its role in integration and coordination of multiple morphogen stimuli, malfunctioning of parafibromin, whether gain-of-function or loss-of-function, may be associated with the development of various human disorders including developmental and malignant diseases.

## Methods

### Cell culture and transfections

HEK293T human embryonic kidney cells (ATCC CRL3216), A549 lung adenocarcinoma-derived epithelial cells (Japanese Cancer Research Resources Bank), MCF7 human mammary carcinoma-derived epithelial cells (ATCC HTB22) and *Hrpt2*^*flox/flox*^*/CAG-CreER* MEF cells were cultured in Dulbecco's modified Eagle's medium (DMEM) supplemented with 10% fetal bovine serum (FBS). AGS human gastric carcinoma-derived cells (ATCC CRL1739) and MKN28 human gastric carcinoma-derived cells (Japanese Cancer Research Resources Bank) were cultured in RPMI 1640 medium with 10% FBS. SKES human Ewing sarcoma cells (ATCC HTB86) were cultured in McCoy's 5A medium (Gibco) supplemented with 15% FBS. To activate endogenous Wnt signalling, HEK293T cells were treated with 25 mM LiCl, which inhibits GSK3β and prevents β-catenin degradation. To inhibit endogenous Notch signalling, cells were incubated with medium containing 100 μM *N*-[*N*-(3,5-diflurophenylacetate)-L-alanyl]-(*S*)-phenylglycine *t*-butyl ester DAPT (Millipore) for 48 h. Transient transfection was carried out using Lipofectamine reagent (Invitrogen) with Plus reagent (Invitrogen) for HEK293T and MKN28 cells, and using Lipofectamine 2000 reagent (Invitrogen) for MCF7, A549, and AGS cells. *Hrpt2*^*flox/flox*^*/CAG-CreER* MEFs were treated with 500 nM 4-Hydroxytamoxifen (4-OHT, Sigma) for 72 h to conditionally delete *Hrpt2* alleles.

### Plasmids

The expression vectors for N-terminally Flag-tagged wild-type parafibromin, phospho-resistant Y290/293/315F parafibromin (PR-parafibromin), β-catenin-binding site-deleted parafibromin (Δβ-parafibromin) and Myc-tagged human SHP2 were described previously^21^. The cDNAs encoding human GLI1 and mouse Notch1 intracellular domain (amino-acid sequences of 1,744–2,183) were cloned into the pcDNA3 vector (Invitrogen). The cDNA encoding human PTK6 was cloned into the pRc/CMV vector (Invitrogen). The expression vector for hemagglutinin (HA)-tagged β-catenin S33Y was generated from the expression vector for HA-β-catenin by introducing a point mutation (S33Y) using a Chameleon site-directed mutagenesis kit (Stratagene). Wnt-responsive Top-tk and Notch-responsive pTP1 luciferase reporter plasmids have been described previously^52^,^53^. Hedgehog luciferase reporter plasmid GLI-BS was kindly provided by Dr M. Ishibashi (Chiba University).

### Antibodies

Anti-Flag (M2, SIGMA, 1:2000), anti-HA (3F10, Roche and 16B12, COVANCE, 1:1000), anti-Myc (9E10, Santa Cruz, 1:1000), anti-Actin (C-11, Santa Cruz, 1:500), anti-parafibromin (A300-170A and A300-171A, Bethyl, 1:1000), anti-NICD (#2421, Cell signalling, 1:1,000; the antibody recognizes Notch protein only when it is released by cleavage between Gly1743 and Val1744 of murine Notch1 (equivalent to Gly1753 and Val1754 of human NOTCH1), as shown in Supplementary Fig. 2a,b), anti-β-catenin (H102, Santa Cruz and 610153, BD Biosciences, 1:1,000), anti-HES1 (H-20, Santa Cruz, 1:1,000) and anti-Gli1 (H300, Santa Cruz and L42B10, Cell Signaling, 1:1,000) antibodies were used as primary antibodies for immunoblotting and immunostaining. For immunohistochemistry, anti-β-catenin (H102, Santa Cruz), anti-CD44 (550538, BD Biosciences), anti-Sox9 (AB5535, Millipore), anti-Ki-67 (TEC3, Dako), anti-Hes1 (H-20, Santa Cruz), anti-Villin (C-19, Santa Cruz), anti-parafibromin (A300-170A, Bethyl) and anti-Ptk6 (C-17, Santa Cruz) antibodies were used as primary antibodies.

### Immunoprecipitation and immunoblotting

Cells were collected and lysed in lysis buffer containing 250 mM NaCl, 50 mM Tris-HCl (pH 8.0), 5 mM EDTA, 0.5% NP-40, 2 mM Na_3_VO_4_, 10 mg ml^−1^ leupeptin, 10 mg ml^−1^ trypsin inhibitor, 10 mg ml^−1^ aprotinin and 2 mM phenylmethylsulfonyl fluoride. For immunoprecipitation, cell lysates were incubated with respective antibodies and protein G-beads (GE Healthcare). The beads were then washed six times with the lysis buffer, and the immune complex was eluted with SDS–polyacrylamide gel electrophoresis (PAGE) sample buffer. The lysates and immunoprecipitates were subjected to SDS–PAGE followed by immunoblotting. Proteins were transferred to a polyvinylidene difluoride membrane filter (Millipore) and visualized using western blot chemiluminescence reagent (Perkin-Elmer Life Sciences). The obtained chemiluminescence was exposed to X-ray film (GE Healthcare). Uncropped images for immunoblots are shown in Supplementary Fig. 8.

### Luciferase reporter assay

Luciferase activities were measured using the dual luciferase reporter assay kit (Promega) according to the manufacturer's protocol. pRL/CMV- and pRL/TK-luciferase reporter plasmids were used as a second reporter. For Notch reporter assays, HEK293T cells were seeded in 12-well plates (1.5 × 10^5^ cells per well) and transfected with the reporter plasmids using Lipofectamine reagent (Invitrogen) with Plus reagent (Invitrogen). Cells were collected 72 h after transfection using the Passive lysis buffer (Promega). Endogenous or NICD-induced Notch signal activity was then measured using the dual luciferase reporter assay kit (Promega). The data were obtained by analysing triplicated samples each prepared from three independent experiments. The pcDNA3 empty vector was used for a control plasmid.

### Immunostaining

HEK293T cells, MKN28 cells and intestinal organoids were washed with PBS and fixed with 4% paraformaldehyde. Samples were then permeabilized with 0.1% TritonX-100 and incubated in 1% BSA/PBS, followed by first antibody treatment. Fluorescent images were obtained using TCS-SPE (Leica) and FLUOVIEW FV1200 (Olympus) confocal microscope systems.

### Mice

*Hrpt2*^*flox/flox*^ mice (kindly provided by B. Williams, Van Andel Institute)^33^, *CAG-CreER* transgenic mice (The Jackson Laboratory, Stock No: 004453)^54^ and *Villin-Cre* transgenic mice (The Jackson Laboratory, Stock No: 004586)^55^ were used for experiments. *Hrpt2*^*flox/flox*^ mice were crossed with *CAG-CreER* transgenic mice and with *Villin-Cre* transgenic mice to generate *Hrpt2*^*flox/flox*^/*CAG-CreER* and *Hrpt2*^*flox/flox*^/*Villin-Cre* mice, respectively. At ages of 4–6 weeks, male *Hrpt2*^*flox/flox*^/*CAG-CreER* mice and the control littermates were injected intraperitoneally daily with tamoxifen dissolved in corn oil (4 mg per 40 g body weight). Mice were killed and examined at 5 days after exposure to tamoxifen. To generate *Ptk6* knockout mice using CRISPR/Cas9-mediated genome editing^29^,^30^, we constructed pDR274 plasmids (Addgene #42250) encoding single-guide RNA (sgRNA) targeting 20 nt sequence in the *Ptk6* allele as shown in Supplementary Fig. 5b. The sgRNA was transcribed *in vitro* using the DraI-digested vector as a template and the MEGAshortscript T7 kit (Ambion) according to manufacture's instruction. The synthesized sgRNA was then purified using mini Quick Spin Column (Roche) according to the manufacturer's instruction. The *Cas9* mRNA was transcribed *in vitro* using Pme1-digested Cas9 expression vector as a template and the MessageMaxTM T7 mRNA transcription kit (Cellscript) according to the manufacturer's instruction. Following completion of the poly(A) tailing reaction was performed using A-Plus Poly(A) Polymerase Tailing kit (Cellscript) according to the manufacturer's instruction. *Cas9* mRNA was then purified using MEGAclear kit (Ambion) according to the manufacturer's instruction. An amount of 100 ng μl^−1^ Cas9 mRNA and 50 ng μl^−1^ of sgRNA were mixed and microinjected into the cytoplasm of C57BL/6J (CLEA Japan, Inc.) fertilized eggs. Obtained founder (F_0_) mice were genotyped and crossed with C57BL/6J mice to obtain F_1_ mice. F_1_ mice were then intercrossed to obtain homogenous *Ptk6* knockout (*Ptk6*^*−/−*^) mice. At ages of 3 weeks, female *Ptk6*^*−/−*^ mice and the control littermates were killed and used for the experiments. All animals were treated and maintained in accordance with the protocol approved by the Ethics Committees for Animal Experiment at the Graduate School of Medicine, The University of Tokyo.

### Immunohistochemistry

Freshly sampled mouse tissues were flushed with ice-cold PBS and fixed by incubation in 4% paraformaldehyde in PBS overnight at 4 °C. Fixed tissues were dehydrated, embedded in paraffin, and sectioned. The sections were de-waxed and rehydrated. Endogenous peroxidase activity was blocked by incubation in 0.3% hydrogen peroxide in methanol for 30 min at room temperature. Antigen retrieval was performed by boiling for 15 min in citrate buffer pH 6.0 or Tris-EDTA pH 9.0. Tissues were incubated overnight at 4 °C with the following primary antibodies: anti-β-catenin (H102, Santa Cruz, 1:500), anti-CD44 (550538, BD Biosciences, 1:500), anti-Sox9 (AB5535, Millipore, 1:5,000), anti-Ki-67 (TEC3, Dako, 1:25), Gli1 (H300, Santa Cruz, 1:125), anti-HES1 (H-20, Santa Cruz, 1:50), anti-Gapdh (G9545, Sigma, 1:100), anti-SHP2 (B-1, Santa Cruz, 1:1,000), anti-Villin (C-19, Santa Cruz, 1:50), anti-parafibromin (A300-170A, Bethyl, 1:500), and anti-Ptk6 (C-17, Santa Cruz, 1:500). Staining was performed using the Vectastain ABC-Elite kit according to the manufacturer's instructions, except for Sox9, which was detected using an anti-rabbit Alexa-Fluor 546 (Invitrogen). For detection of apoptosis, terminal deoxynucleotidyl-transferase-mediated biotin-dUTP nick end labelling (TUNEL) staining was performed using *In situ* Apoptosis Detection Kit (MK500, TaKaRa) according to the manufacturer's protocol. Rat mammary gland tissue slides (MK504, TaKaRa) were used as a positive control for TUNEL staining.

### Crypt isolation and organoid culture

For isolation of the small intestinal crypt, mice were euthanatized and isolated small intestines were opened longitudinally using small scissors. The tissues were washed with cold PBS and the villi were scraped using a haemacytometer coverslip. The tissues were then chopped into small 2–4 mm pieces, washed with cold PBS and incubated with PBS containing 2 mM EDTA for 30 min. After incubation, the supernatant was removed and the tissue fragments were vigorously suspended with 10 ml cold PBS. The supernatant (enriched for crypts) was collected and centrifuged at 200*g*. for 3 min. The pelleted crypts were then mixed with Matrigel (BD Bioscience), plated in 48 well plates, and cultured in 400 μl of crypt culture medium^31^. For gene deletion, 500 nM 4-OHT was added to the culture medium and maintained until the time of observations. To examine the effect of the BMP signal, organoids were treated with or without recombinant human BMP4 (100 ng ml^−1^, R&D) for 42 h.

### Quantitative PCR

Total RNA was extracted from cells using TRIzol reagent (Invitrogen) and subjected to reverse transcription by SuperScriptII (Invitrogen). cDNAs were analysed by the StepOnePlus Real-Time PCR System (Applied Biosystems) using SYBR Premix Ex Taq (TaKaRa). *TBP* or *Gapdh* was used to normalize input. Following primers were used;

Human *HES1*, 5′-CCAAAGACAGCATCTGAGCA-3′ and 5′-CCGCGAGCTATCTTTCTTCA-3′;

Human *HEY1*, 5′-GAGACCATCGAGGTGGAGAA-3′ and 5′-TCGGCGCTTCTCAATTATTC-3′;

Human *TBP*, 5′-GCTCACCCACCAACAATTTAGTAG-3′ and 5′-CTGCTCTGACTTTAGCACCTGTTA-3′

Mouse *Hes1*, 5′-TCAACACGACACCGGATAAA-3′ and 5′-TCAGCTGGCTCAGACTTTCA-3′;

Mouse *Hey1*, 5′-TCGGCTCTAGGTTCCATGTC-3′ and 5′-CTGGGTACCAGCCTTCTCAG-3′;

Mouse *Gapdh*, 5′-ACCACAGTCCATGCCATCAC-3′ and 5′-TCCACCACCCTGTTGCTGTA-3′.

### Proximity ligation assay

PLA was performed with a Duolink *In situ* PLA Kit (Olink Bioscience) according to the manufacturer's protocol.

### Preparation of recombinant proteins

For purification of parafibromin, BL21 (DE3) *E. coli* strain transformed by the pGEX-6P-1/Flag-parafibromin-His plasmid was cultured at 37 °C in LB medium with ampicillin. When OD_600_ reached 0.8, the recombinant protein was inducibly expressed at 18 °C with 100 mM IPTG for 13 h. The obtained *E. coli* was suspended in Ni-1 buffer (20 mM Tris-HCl pH 8.0, 100 mM MgCl_2_, 10 mM imidazole, 0.3 mg ml^−1^ benzamidine) and then the soluble fraction, which was prepared through ultrasonication (Astrason Ultrasonic Processor XL2020), was incubated with Ni-NTA agarose beads (Qiagen) at 4 °C for 1 h. After washing with Ni-2 buffer (20 mM Tris-HCl pH 8.0, 100 mM MgCl_2_, 20 mM imidazole), the recombinant parafibromin was eluted into Ni-3 buffer (20 mM Tris-HCl pH 8.0, 100 mM MgCl_2_, 250 mM imidazole; Ni-affinity column chromatography). The lysate obtained was incubated with Glutathione Sepharose 4B beads (GE Healthcare) for 1 h at 4 °C and then applied to GST-affinity column chromatography, using W1 buffer (25 mM Tris-HCl pH 7.5, 150 mM NaCl, 10 mM β-mercaptoethanol, 1% Triton X-100) and W2 buffer (50 mM Tris-HCl pH 8.0, 150 mM NaCl, 10 mM β-mercaptoethanol) for washing. The recombinant parafibromin was eluted into W2 buffer with 10 mM glutathione and then concentrated via ultrafiltration using Amicon Ultra (Millipore). For generation of tyrosine-phosphorylated recombinant parafibromin (pY-GST-Flag-parafibromin-His), GST-Flag-parafibromin-His was co-expressed together with *v*-Src kinase in *E. coli*. For purification of PTK6, *E. coli* BL21 strain transformed by pGEX-6P-2/PTK6-HA was cultured as described above. When OD_600_ reached 0.8, protein induction was started by adding IPTG (33 mM) and the culture was continued for 16 h at 12 °C. The *E. coli* was suspended in a buffer (25 mM Tris-HCl pH 7.5, 150 mM NaCl, 5 mM EDTA, 10 mM β-mercaptoethanol) and then the soluble fraction was obtained via ultrasonication. The lysate obtained was subjected to GST-affinity column chromatography using W2 buffer containing 20 mM glutathione for elution and was concentrated as mentioned above.

### *In vitro* kinase assay

Recombinant GST-Flag-parafibromin-His (10 pmol) and GST-PTK6-HA (1 pmol) were mixed and incubated at 30 °C for 10 min in 30 μl of kinase buffer (10 mM HEPES, 150 mM NaCl, 2.5 mM DTT, 0.01% Triton X-100, 10 mM MnCl_2_) with or without 200 μM ATP. The *in vitro* phosphorylation was terminated by adding the SDS sample buffer to the reaction mixture. The reaction products were loaded onto an SDS–PAGE gel and then analysed through the immunoblotting using the indicated antibodies.

### Statistics

No statistical methods were used to predetermine sample size. For experiments, a minimum of three samples were chosen as a sample size to ensure adequate power. The experiments were not randomized. The investigators were not blinded to allocation during experiments and outcome assessment. Statistical analysis was performed using either a two-tailed unpaired Student's *t*-test, ANOVA with Bonferroni's *post hoc* test or the Wilcoxon rank sum test.

### Data availability

The data that support the findings of this study are available within the Article and Supplementary Information files, or available from the corresponding author on request.

## Additional information

**How to cite this article:** Kikuchi, I. *et al*. Dephosphorylated parafibromin is a transcriptional coactivator of the Wnt/Hedgehog/Notch pathways. *Nat. Commun.* 7:12887 doi: 10.1038/ncomms12887 (2016).

## Supplementary Material

Supplementary Information Supplementary Figures 1-8
