# Supplementary material for: Dephosphorylated parafibromin is a transcriptional coactivator of the Wnt/Hedgehog/Notch pathways
Source: Nat Commun. 2016 Sep 21;7:12887. doi: 10.1038/ncomms12887 (PMC5036006; doi:10.1038/ncomms12887)
Supplement: Supplementary Information — Supplementary Figures 1-8 [file ncomms12887-s1.pdf]

a

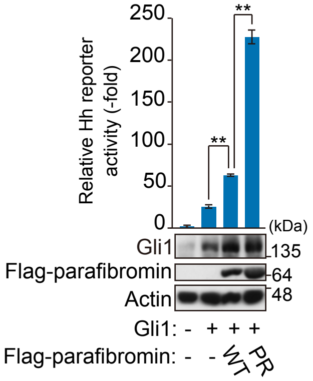

Supplementary Figure 1 Parafibromin transactivates Hedgehog signalling in a tyrosine dephosphorylation-dependent manner. (**a**) Phospho-resistant (PR)-parafibromin shows increased ability to activate Gli1-dependent Hh activation. HEK293T cells were transiently transfected with indicated vectors together with an Hh-responsive reporter plasmid. Total cell lysates were subjected to immunoblotting with indicated antibodies. Relative luciferase activities are shown in graphs with the value in control cells calculated as 1. Error bars indicate means ± SD; n=3. **p<0.01; ANOVA with Bonferroni' s post hoc test.

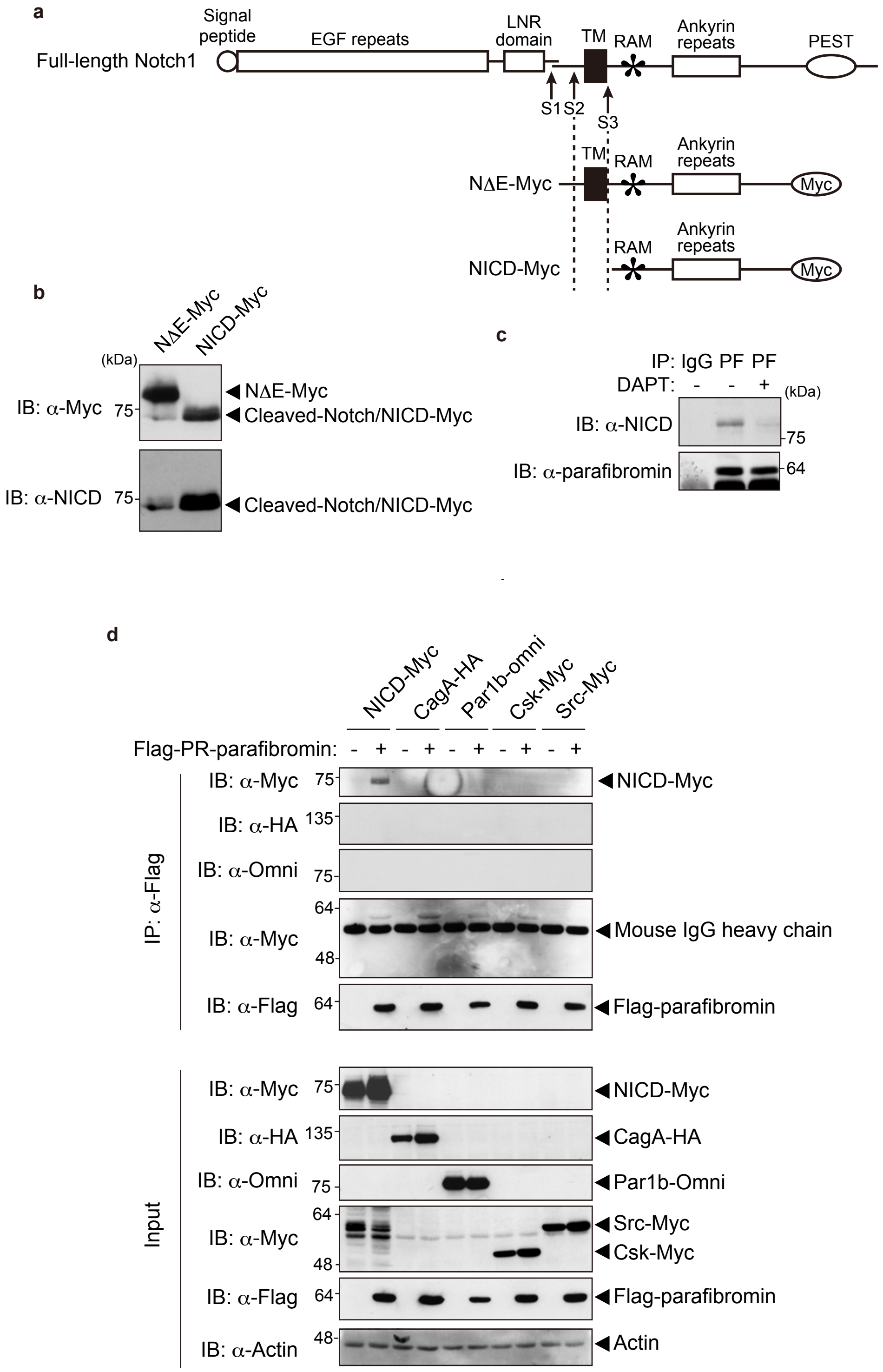
a
Full-length Notch1
Signal peptide
EGF repeats
LNR domain
TM
RAM
Ankyrin repeats
PEST
S1
S2
S3
NΔE-Myc
NICD-Myc
Myc
b
NΔE-Myc
NICD-Myc
(kDa)
IB: α-Myc
75
◀NΔE-Myc
◀Cleaved-Notch/NICD-Myc
IB: α-NICD
75
◀Cleaved-Notch/NICD-Myc
c
IP: IgG PF PF
DAPT: - - +
(kDa)
IB: α-NICD
75
IB: α-parafibromin
64
d
NICD-Myc
CagA-HA
Par1b-omni
Csk-Myc
Src-Myc
Flag-PR-parafibromin: - + - + - + - + - +
IP: α-Flag
IB: α-Myc 75
◀NICD-Myc
IB: α-HA 135
IB: α-Omni 75
IB: α-Myc 64 48
◀Mouse IgG heavy chain
IB: α-Flag 64
◀Flag-parafibromin
Input
IB: α-Myc 75
◀NICD-Myc
IB: α-HA 135
◀CagA-HA
IB: α-Omni 75
◀Par1b-Omni
IB: α-Myc 64 48
◀Src-Myc
◀Csk-Myc
IB: α-Flag 64
◀Flag-parafibromin
IB: α-Actin 48
◀Actin

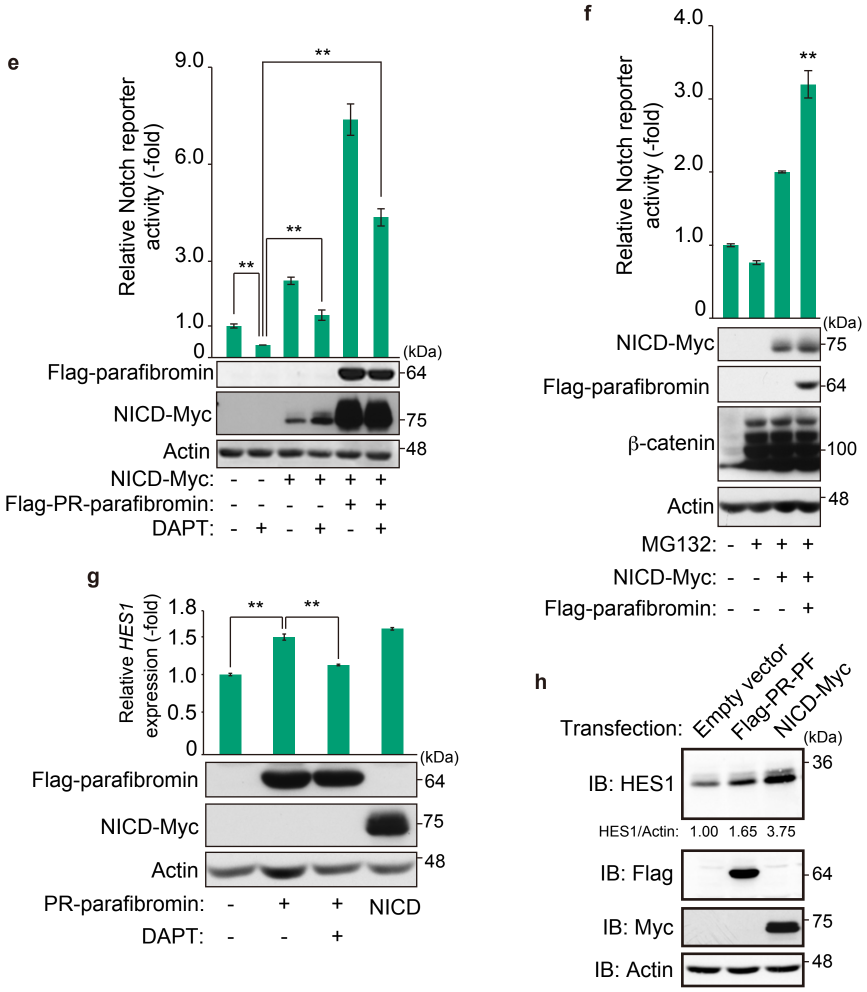

Supplementary Figure 2 Parafibromin acts as a transcriptional co-activator in Notch signalling.

(**a**) A schematic view of Full-length Notch1 and its derivative constructs NΔE-Myc and NICD-Myc used in the present study. γ-secretase-dependent cleavage (S3) occurs at the site between G1743 and V1744 of murine Notch1. (**b**) An anti-NICD antibody used in the present study specifically recognizes cleaved Notch intracellular domain fragment processed from NΔE-Myc. (**c**) A γ-secretase inhibitor (DAPT) treatment abolished the parafibromin-NICD interaction. MCF7 cells were incubated with medium containing 100 μM DAPT for 48 h. Total cell lysates (TCL) prepared were sequentially immunoprecipitated (IP) with an anti-parafibromin antibody and immunoblotted. (**d**) Parafibromin did not show non-specific interaction with biologically irrelevant proteins. HEK293T cells were transfected with the indicated vectors. TCLs were sequentially immunoprecipitated (IP) with an anti-Flag antibody and immunoblotted (IB) with the indicated antibodies. (**e**) Notch-responsive luciferase reporter assays were performed in HEK293T cells (n=3, mean ± SD. **p<0.01; ANOVA with Bonferroni' s post hoc test). Transfected cells were incubated with medium containing 100 μM DAPT for 36 h. (**f**) Parafibromin is capable of activating Notch signal in the presence of proteasome inhibitor. HEK293T cells were transiently co-transfected with indicated vectors together with Notch-responsive reporter plasmid. The cells were incubated with or without 10 μM MG132 for 8 h.TCL were subjected to immunoblotting with indicated antibodies. Relative luciferase activities are shown in graphs with the value in control cells calculated as 1. Error bars indicate means ± SD; n=3. **p<0.01; ANOVA with Bonferroni' s post hoc test. (**g**) MCF7 cells were transfected with the indicated vectors and incubated with medium containing 100 μM DAPT for 48 h. mRNA expression level of *HES1* was measured by RT-qPCR analysis (n=3, mean ± SD. **p<0.01; ANOVA with Bonferroni' s post hoc test). (**h**) MCF7 cells were transfected with the indicated vectors. At 24 h after transfection, cells were lysed and subjected to immunoblotting. Quantification of the band intensity of HES1 relative to Actin is shown.

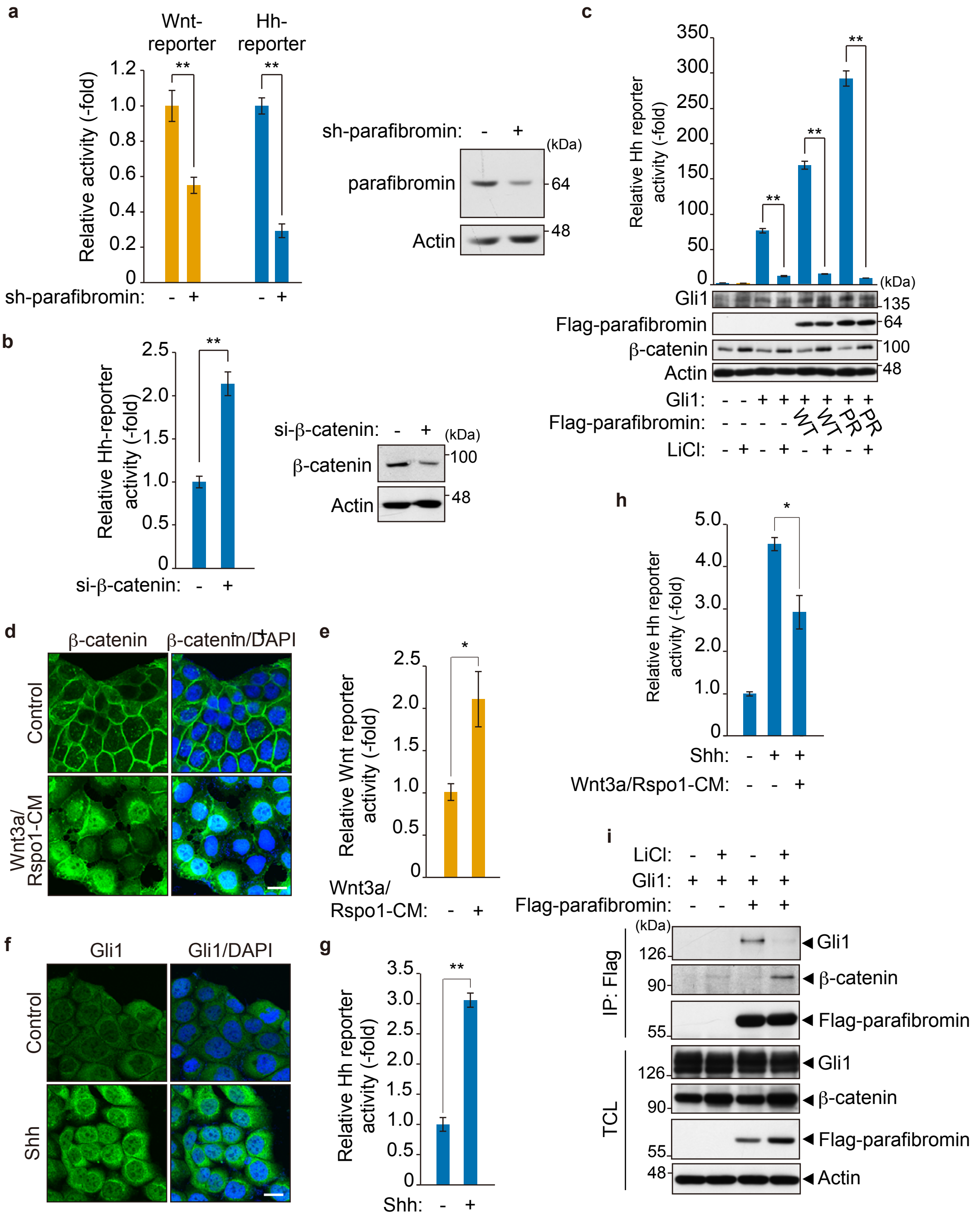

Supplementary Figure 3 Parafibromin activates Wnt signalling and Hedgehog signalling in a mutually exclusive manner. (**a**) Parafibromin knockdown results in reduced endogenous Wnt and Hh signal activity in HEK293T cells. Wnt- or Hh-responsive luciferase reporter assay was performed in HEK293T cells (n=3, mean ± SD. **p<0.01; a two-tailed unpaired Student' s t-test). (**b**) β-catenin knockdown increased endogenous Hh signal activity. Hh-responsive luciferase reporter assay was performed in HEK293T cells (n=3, mean ± SD. **p<0.01; a two-tailed unpaired Student' s t-test). (**c**) LiCl treatment inhibits parafibromin-dependent Hh signal activation. HEK293T cells were transiently co-transfected with indicated vectors together with an Hh-responsive reporter plasmid. Transfected cells were maintained in the presence or absence of 25 mM LiCl. Relative luciferase activities are shown in graphs with the value in control cells calculated as 1. Error bars indicate means ± SD; n=3. **p<0.01; ANOVA with Bonferroni' s post hoc test.

Supplementary Figure 3, continued. (**d,e**) Endogenous Wnt signal activation in MKN28 cells. MKN28 cells were cultured in Wnt3a/Rspo1-conditioned medium and then immunostained with the indicated antibodies (**d**) or subjected to Wnt-responsive luciferase reporter assays (**e**) (n=3, mean ± SD. **p<0.01; a two-tailed unpaired Student' s t-test). A scale bar, 20 μm. (**f,g**) Hh signal activation in MKN28 cells. MKN28 cells were transfected with an Shh expression vector and then immunostained with the indicated antibodies (**f**) or subjected to Hh-responsive luciferase reporter assays (**g**) (n=3, mean ± SD. **p<0.01; a two-tailed unpaired Student' s t-test). A scale bar, 20 μm. (**h**) Endogenous competition between Wnt and Hh signals. MKN28 cells were transfected with an Shh expression vector and cultured with or without Wnt3a/Rspo1-conditioned medium. Hh-responsive luciferase reporter activities were measured (n=3, mean ± SD. **p<0.01; ANOVA with Bonferroni' s post hoc test). (**i**) Endogenous β-catenin competitively suppresses parafibromin/Gli1 interaction. HEK293T cells were transiently transfected with the indicated vectors. Transfected cells were maintained in the presence of 25 mM NaCl or LiCl. Total cell lysates (TCL) were immunoprecipitated with anti-Flag antibody and the immunoprecipitates (IP) were immunoblotted with the indicated antibodies.

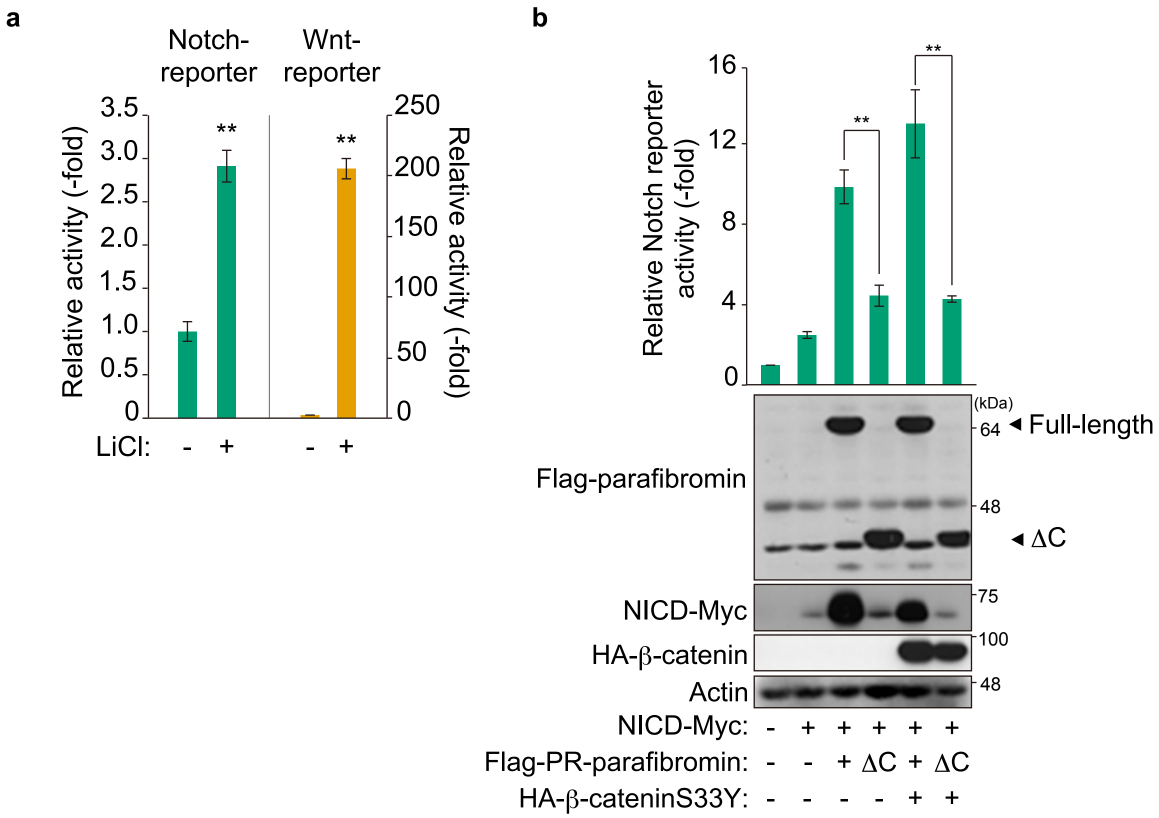

Supplementary Figure 4 Parafibromin activates Wnt and Notch signals in a cooperative fashion. (**a**) Wnt- or Notch-responsive luciferase reporter assay was performed in HEK293T cells treated with 25 mM LiCl for 24 h (n=3, mean ± SD. **p<0.01; a two-tailed unpaired Student' s t-test). (**b**) Notch-responsive luciferase reporter assays were performed in HEK293T cells transfected with the indicated vectors (n=3, mean ± SD. **p<0.01; ANOVA with Bonferroni' s post hoc test).

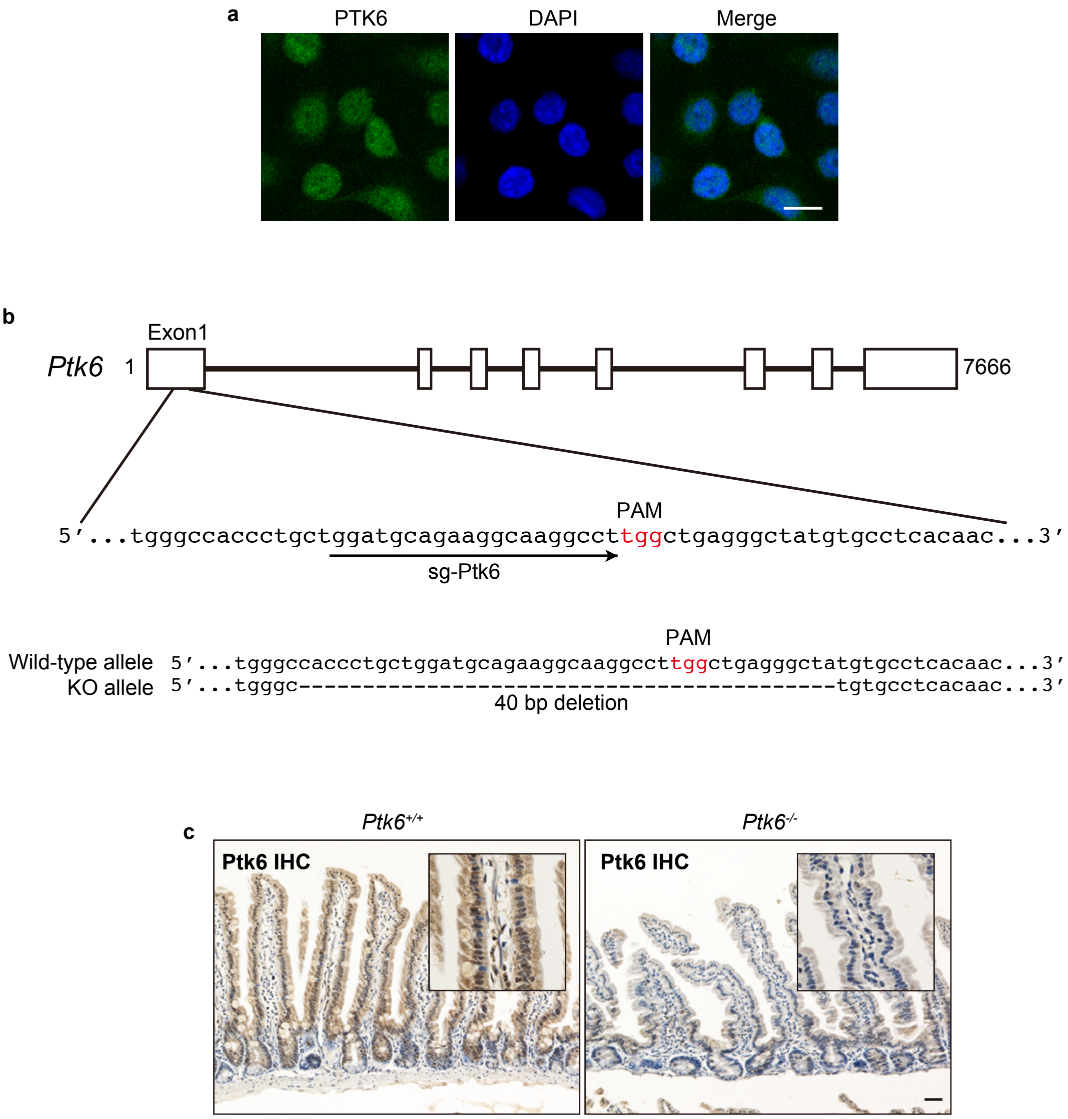

Supplementary Figure 5 PTK6 phosphorylates parafibromin and impairs its scaffold function.

(**a**) Localization of endogenous PTK6 in AGS cells. AGS cells were subjected to immunostaining with an anti-PTK6 antibody. Nuclei were visualized by DAPI staining. A scale bar, 20 μm. (**b**) Schematic depiction of the *Ptk6* locus in *Ptk6* knockout mice generated by CRISPR/Cas9-mediated genome editing. The sequence (downstream of start codon in Exon1) targeted by the small guide RNA (sg-Ptk6) is indicated by the black arrow. The protospacer-adjacent motif (PAM) sequence is depicted in red. Sequences of the wild-type (WT) and targeted (KO) *Ptk6* alleles are also indicated. The KO allele carries 40 bp deletion that causes frameshift mutation and loss of Ptk6 expression. (c) Immunohistochemistry of the mouse small intestine of 3 weeks old *Ptk6* KO (*Ptk6*$^{-/-}$) mice confirmed the specificity of an anti-Ptk6 antibody used in the present study. A scale bar, 20 μm. Please note that the intestine of 3-week-old *Ptk6*$^{-/-}$ mice shown here does' t show enhanced villus length and crypt depth, phenotypes previously reported in the adult (older than 8-week-old) *Ptk6*$^{-/-}$ KO intestine[44]. Given that Ptk6 expression is developmentally regulated and exhibits the highest level in the adult intestine[44], the difference is most likely due to the difference in the age of mice used for analysis.

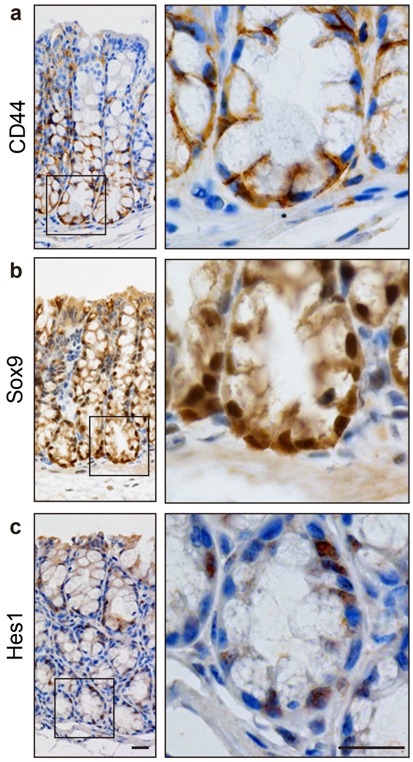

Supplementary Figure 6 Expression of Wnt and Notch targets in intestinal crypts. (**a**) CD44, (**b**) Sox9, and (**c**) Hes1 immunohistochemistry of mouse colonic epithelial tissues. The right panels show higher-magnification images of crypt bottom regions. Scale bars, 20 μm.

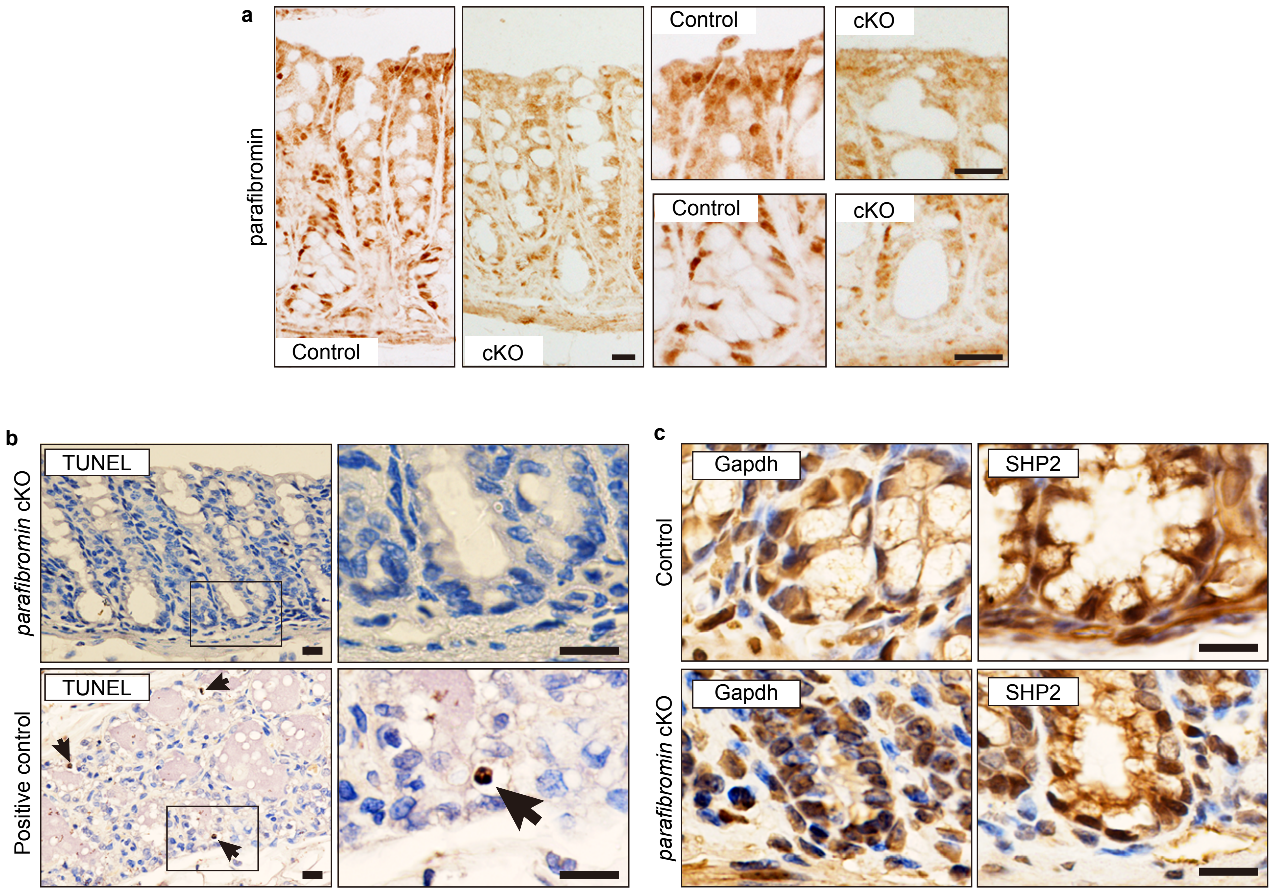

Supplementary Figure 7 Loss of parafibromin leads to disorganization of the intestinal epithelium. (**a-c**) Parafibromin immunohistochemistry (**a**), TUNEL staining (**b**), and Gapdh and SHP2 immunohistochemistry (**c**) of colonic epithelial tissues from control and *Hrpt2*$^{flox/flox}$*/CAG-CreER* conditional knockout (cKO) mice at 5 days after tamoxifen injection. For TUNEL staining in (**b**), a rat mammary gland tissue was used as a positive control. Scale bars, 20 µm.

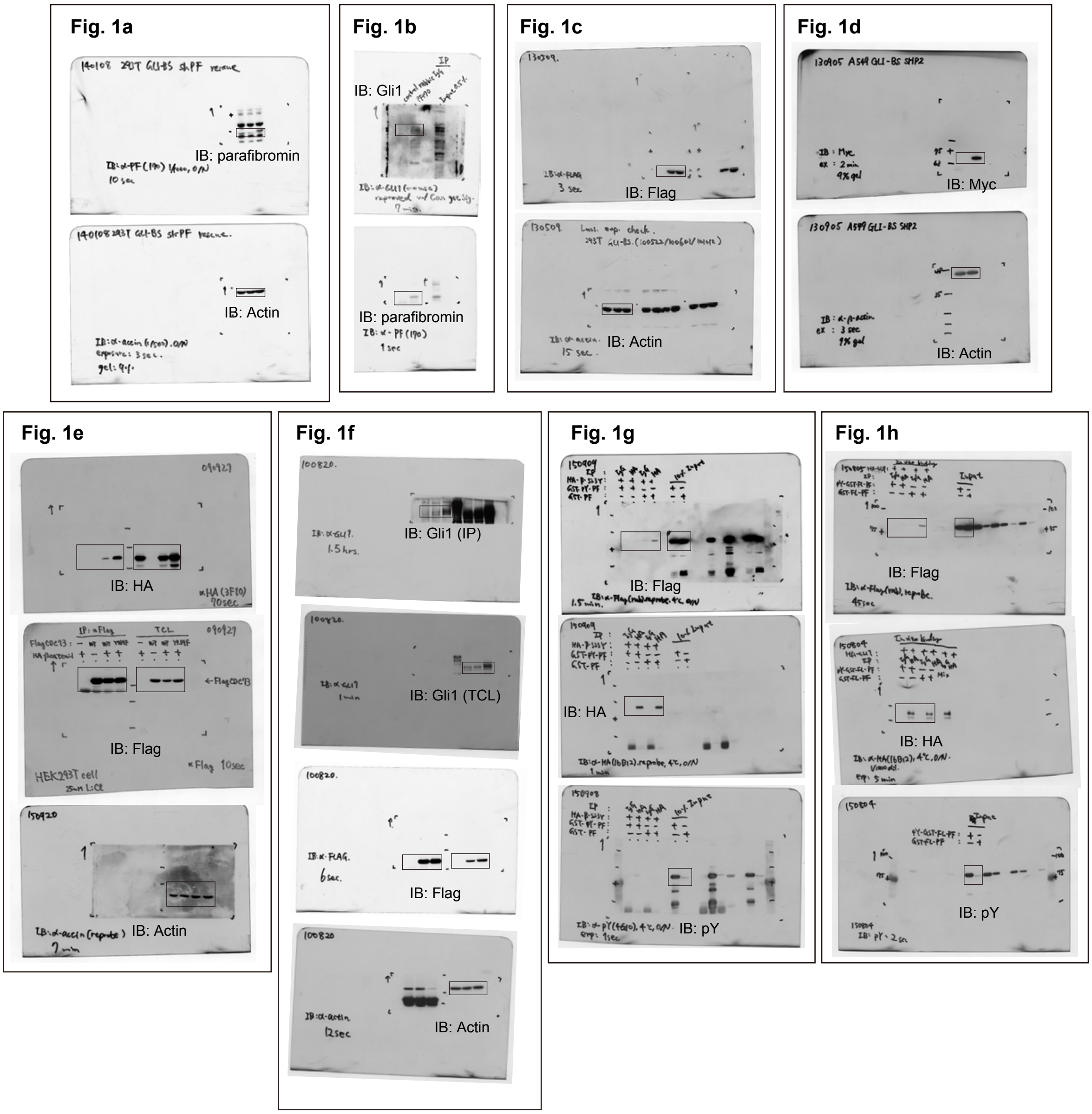

Supplementary Figure 8 Full scans of original blots in the paper. Panels corresponding to each figure in the paper are indicated.

Fig. 2a

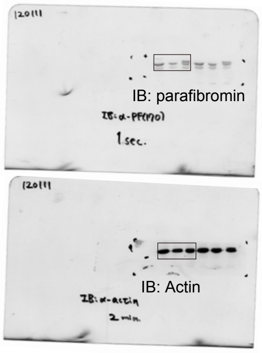

Fig. 2b

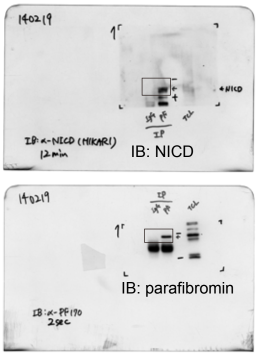

Fig. 2c

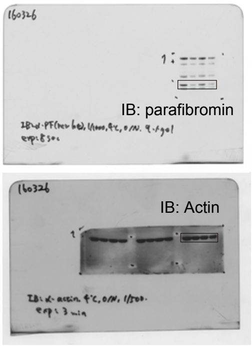

Fig. 2d

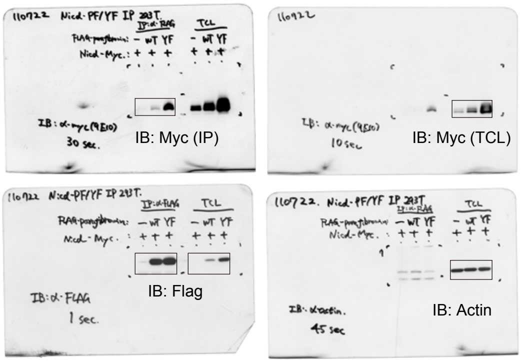

Fig. 2e

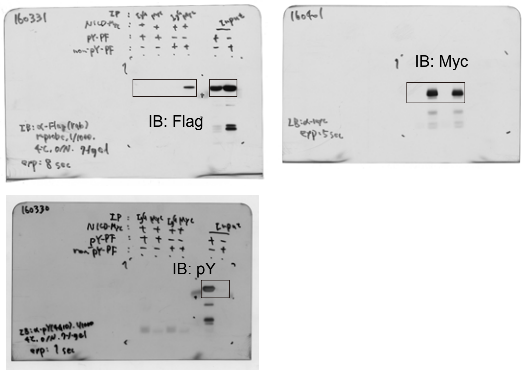

Fig. 2f

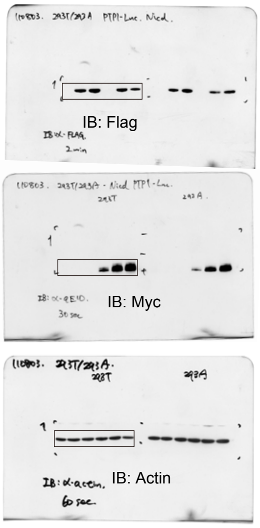

Fig. 2g

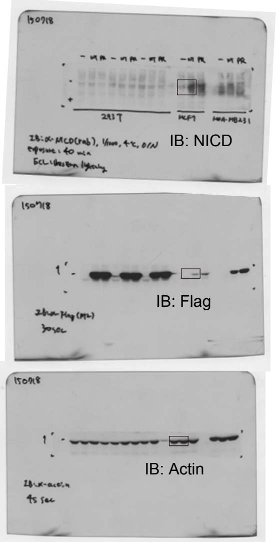

Fig. 2h

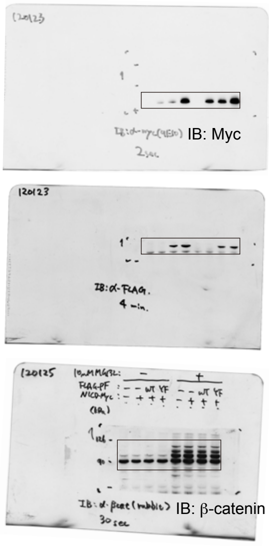

Fig. 2j

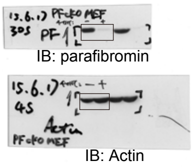

Supplementary Figure 8 continued Full scans of original blots in the paper. Panels corresponding to each figure in the paper are indicated.

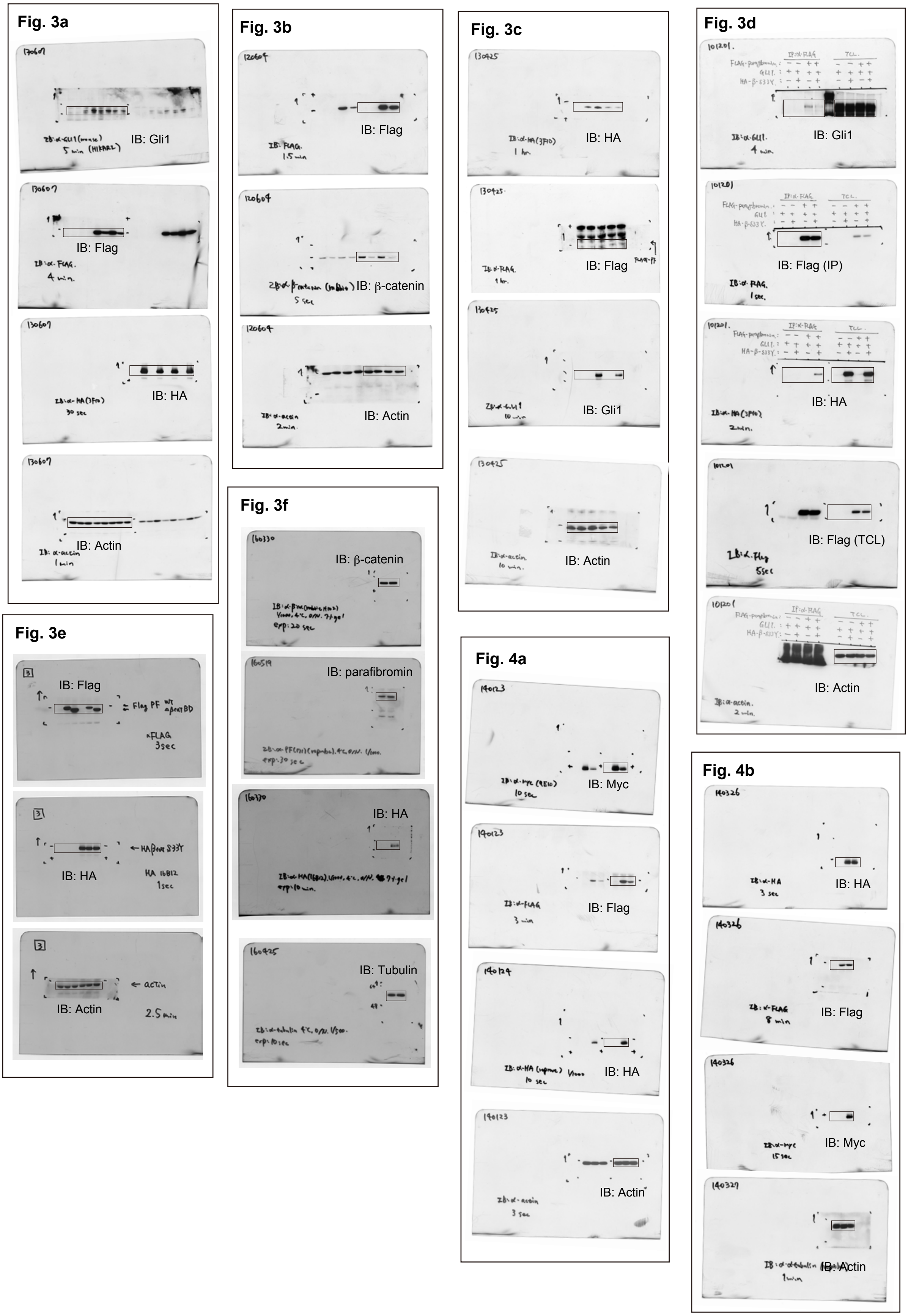

Supplementary Figure 8 continued Full scans of original blots in the paper. Panels corresponding to each figure in the paper are indicated.

Fig. 4c

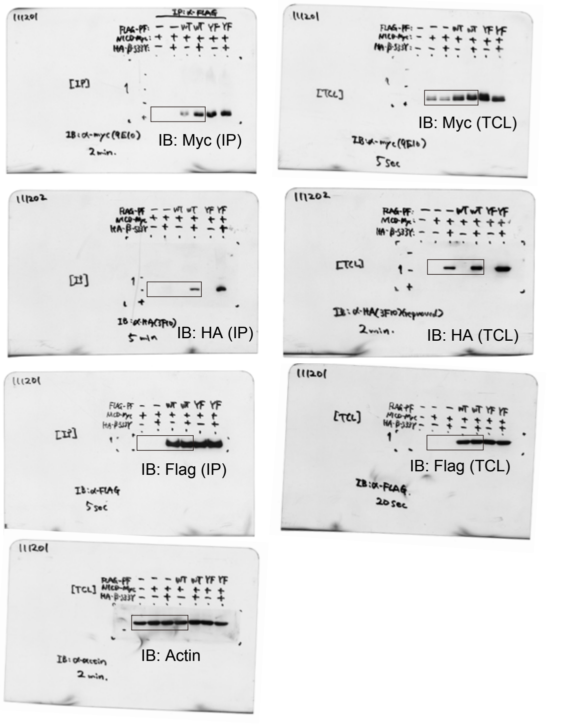

Fig. 4d

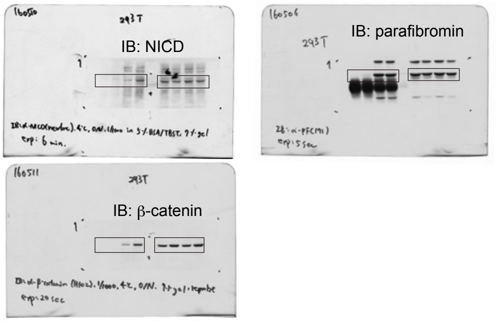

Fig. 4e

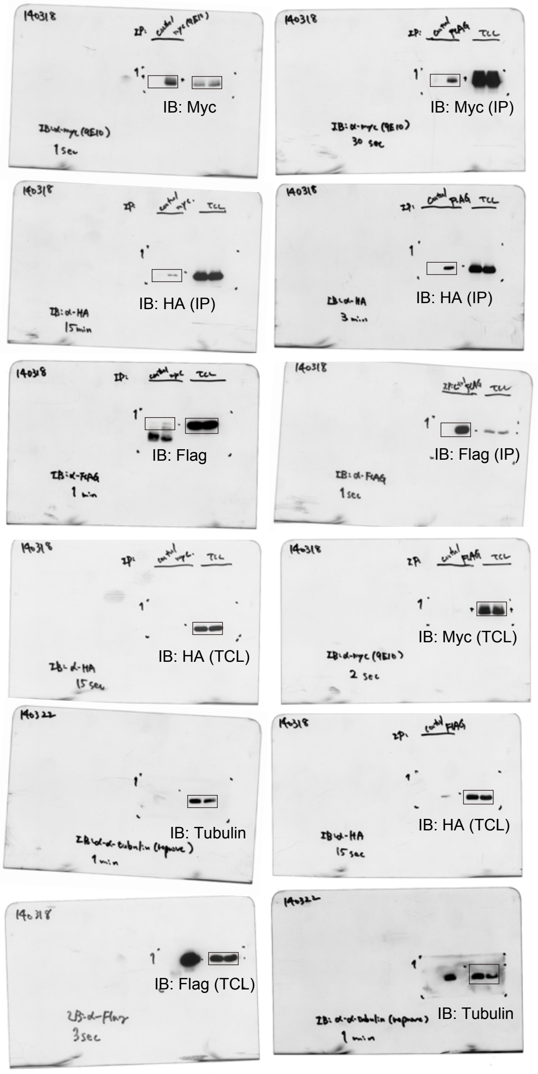

Fig. 4g

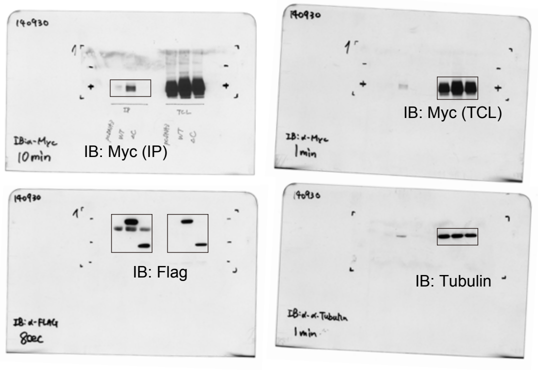

Supplementary Figure 8 continued Full scans of original blots in the paper. Panels corresponding to each figure in the paper are indicated.

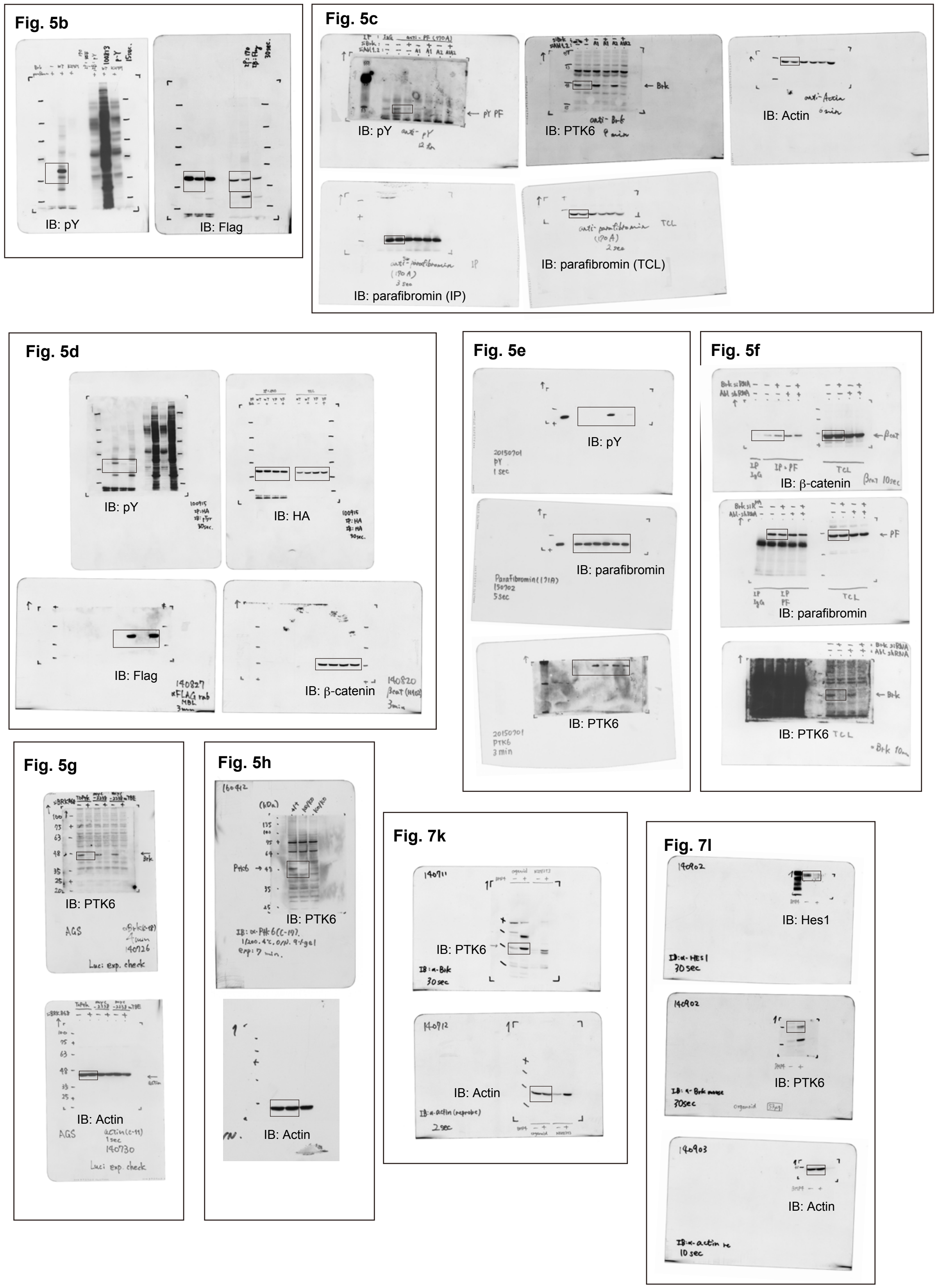

Supplementary Figure 8 continued Full scans of original blots in the paper. Panels corresponding to each figure in the paper are indicated.

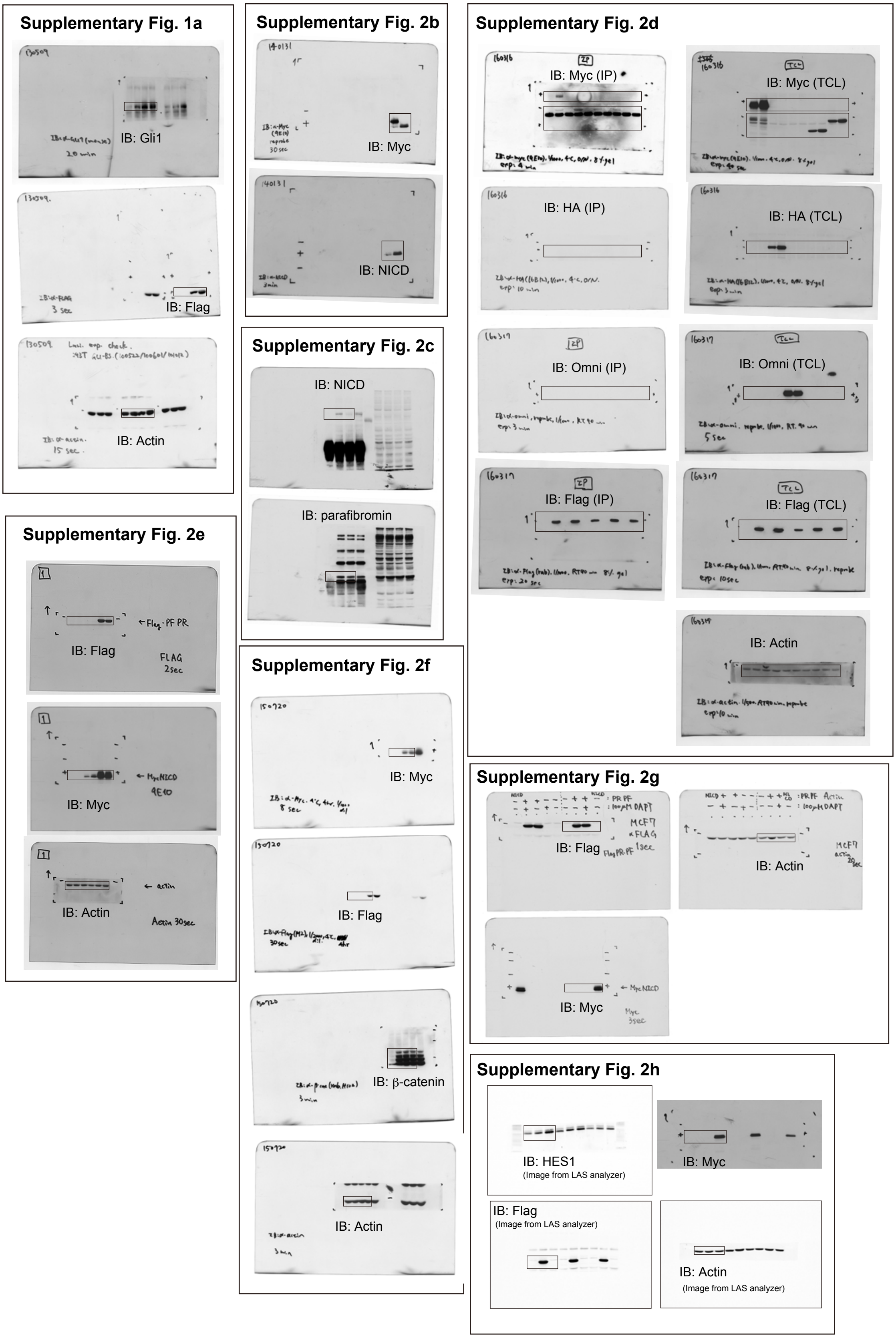

Supplementary Figure 8 continued Full scans of original blots in the paper. Panels corresponding to each figure in the paper are indicated.

Supplementary Fig. 3a

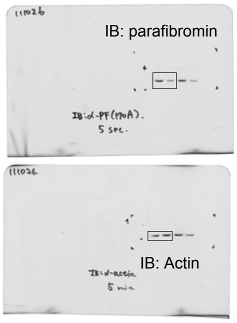

Supplementary Fig. 3b

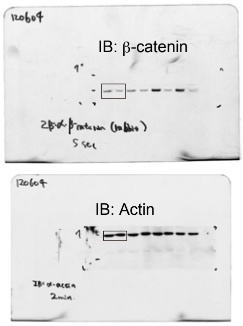

Supplementary Fig. 3c

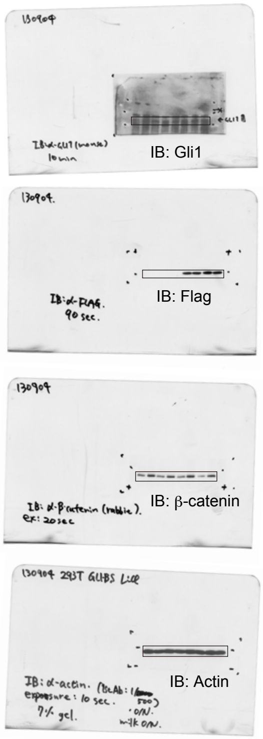

Supplementary Fig. 3i

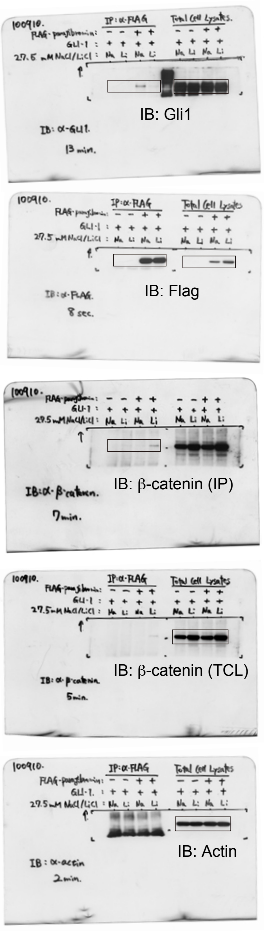

Supplementary Fig. 4b

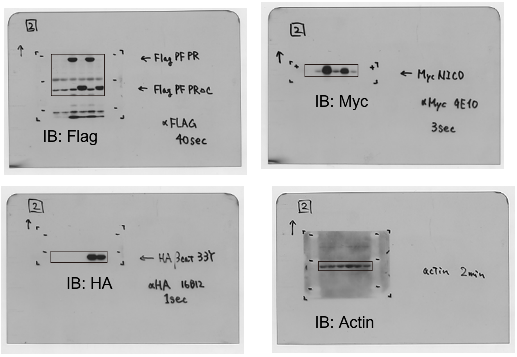

Supplementary Figure 8 continued Full scans of original blots in the paper. Panels corresponding to each figure in the paper are indicated.
